# Boosting intracellular sodium selectively kills hepatocarcinoma cells and induces hepatocellular carcinoma tumor shrinkage in mice

**DOI:** 10.1038/s42003-023-04946-4

**Published:** 2023-05-29

**Authors:** Nausicaa Clemente, Simona Baroni, Simone Fiorilla, Francesco Tasso, Simone Reano, Chiara Borsotti, Maria Rosaria Ruggiero, Elisa Alchera, Marco Corrazzari, Gillian Walker, Antonia Follenzi, Simonetta Geninatti Crich, Rita Carini

**Affiliations:** 1 Department of Health Science Università del Piemonte Orientale, Via Solaroli, 17, 28100 Novara, Italy; 2 Department of Molecular Biotechnology and Health Sciences, University of Torino, Via Nizza, 52, 10126 Torino, Italy; 3 Department of Department of Translational Medicine, Unit of Muscle Biology, Università del Piemonte Orientale, Via Solaroli, 17, 28100 Novara, Italy; 4 Division of Experimental Oncology/Unit of Urology, URI, IRCCS, Ospedale San Raffaele, Milan, Italy; 5 Department of Health Science and Interdisciplinary Research Center of Autoimmune Disease (IRCAD), Università del Piemonte Orientale, Via Solaroli, 17, 28100 Novara, Italy

**Keywords:** Hepatocytes, Targeted therapies

## Abstract

Pharmacological treatments for advanced hepatocellular carcinoma (HCC) have a partial efficacy. Augmented Na^+^ content and water retention are observed in human cancers and offer unexplored targets for anticancer therapies. Na^+^ levels are evaluated upon treatments with the antibiotic cation ionophore Monensin by fluorimetry, ICP-MS, ^23^Na-MRI, NMR relaxometry, confocal or time-lapse analysis related to energy production, water fluxes and cell death, employing both murine and human HCC cell lines, primary murine hepatocytes, or HCC allografts in NSG mice. Na^+^ levels of HCC cells and tissue are 8-10 times higher than that of healthy hepatocytes and livers. Monensin further increases Na^+^ levels in HCC cells and in HCC allografts but not in primary hepatocytes and in normal hepatic and extrahepatic tissue. The Na^+^ increase is associated with energy depletion, mitochondrial Na^+^ load and inhibition of O_2_ consumption. The Na^+^ increase causes an enhancement of the intracellular water lifetime and death of HCC cells, and a regression and necrosis of allograft tumors, without affecting the proliferating activity of either HCCs or healthy tissues. These observations indicate that HCC cells are, unlike healthy cells, energetically incapable of compensating and surviving a pharmacologically induced Na^+^ load, highlighting Na^+^ homeostasis as druggable target for HCC therapy.

## Abstract (web summary)

The ionophore monensin is shown to have cancer-selective cytotoxic action by selectively increasing the sodium content in cultured hepatocellular carcinoma cells (HCC) and allografts, highlighting the sensitivity of HCC cells to pharmacologically induced Na+ load.

## Introduction

Hepatocarcinoma (HCC) is the fourth leading cause of cancer-related deaths^1^, with its incidence expected to increase in the next decades^2^. HCC is often detected in intermediate and advanced stages and the mainstay treatment in these phases is a systemic therapy with tyrosine kinase inhibitors^2, 3^. This approach improves the short-term survival of patients, but rarely does it assure a complete remission, and may in turn induce harmful side effects on normal tissues^2,3^.

The search for alternative therapeutic options, such as targeting specific features of cancer cells, is critical. Cancer-specific features are very few, however, one which is virtually common to most cancers is a reverse pH gradient with an intracellular alkalization and concomitant extracellular acidification^4,5^. Intracellular alkaline pH relies on a potentiated response to the rise of acid species generated by the activation of glycolysis, which is the main source of energy in cancer cells even in presence of oxygen (Warburg effect). Intracellular alkalosis is due to the increased expression and activation of pH regulatory proteins, amongst which are the Na^+^-dependent transporters which operate favoring a net Na^+^ influx from the extracellular space into the cytosol^4,5^.

In early studies with non-cancerous primary rodent hepatocytes (HPs), we investigated the role of altered intracellular Na^+^ concentrations ([Na^+^]_i_) with death and resistance to death. We found that a progressive Na^+^ load preceded HP death induced by toxic conditions that affected mitochondrial activity and energy production, and that the prevention of a Na^+^ increase delayed the appearance of cell death^6,7^. The irreversible alteration of Na^+^ homeostasis caused a deregulation of the hepatocellular volume decrease mechanisms, and eventually caused the death of the HPs^6–8^.

The significance of [Na^+^]_i_ variations in cancer cell viability is, to date, unknown and the data available on Na^+^ homeostasis in cancer is limited. Pioneering studies employing energy-dispersive X-ray microanalysis have, however, reported a significant increase of intracellular Na^+^ in tumor rodent cells when compared to non-cancerous primary cells^9^. More recently, non-invasive ^23^Na-Magnetic Resonance Imaging (^23^Na-MRI) analysis of human malignant gliomas, breast and prostate tumors, confirmed a higher concentration of Na^+^ compared to the surrounding normal tissues^10–13^. Moreover, MRI^14,15^ and Fast-Field-Cycling Nuclear Magnetic Resonance (NMR) relaxometric studies^16–18^, showed a correlation between the increased cellular water molecule efflux rate constant (*k*_io_) and cancer aggressiveness. As such, the increase of the [Na^+^]_i_ and the water fluxes through the cell membrane have only recently been considered as novel cancer biomarkers valuable for both diagnostic and prognostic purposes^10–18^. They also represent two entirely unexplored targets for cancer therapy.

Monensin is an FDA approved antibiotic for veterinary use, and it is broadly used as an effective and safe feed ingredient with coccidiostatic activity. Besides its veterinary usage, numerous preclinical studies have clearly established that Monensin displays potent anticancer effects in extrahepatic tumors and tumor cells^19,20^. Monensin has been shown to induce apoptosis and to inhibit in vitro proliferation of numerous types of cancer cells including those displaying a multidrug resistance^19^, and to markedly reduce the growth in vivo of xenogenic extra-hepatic tumors^20–24^.

To date, the main chemical property of Monensin has never been investigated in relation to its cancer effects. Monensin is, in fact, a polyether cation ionophore capable of reversibly binding Na^+^ and transporting it across the cell membrane along the concentration gradient^19^. Thus, Monensin can passively move Na^+^ from the extracellular space ([Na^+^]_e_ ≃ 145 mM) to the cytoplasm ([Na^+^]_i_ ≃ 15 mM) inducing a net entry of Na^+^ into the cells^19^.

On the basis of our previous observations with primary rodent HPs and on the emerging evidence of a higher Na^+^ level and osmotic pressure in cancer tissues, in the present study we investigated the hypothesis that cancer cells owing to a higher intracellular Na^+^ concentration in contrast to healthy cells, are selectively unable to compensate and survive a further Na^+^ load induced by the pharmacological treatment of the Na^+^-ionophore, Monensin. To this purpose we evaluated Na^+^ level in hepatocarcinoma cells and HCC allografts and investigated their sensitivity to a forced Na^+^ influx induced by the Na^+^ ionophore in comparison to healthy primary HPs and normal tissues.

## Results

### Na^+^-dependent HCC cell killing, and energy loss induced by the Na^+^ ionophore Monensin

The effects of Monensin on cell toxicity and on Na^+^ intracellular variations were evaluated in murine (C1C7) and human (HepG2) hepatocarcinoma (HCC) cell lines, as well as in primary mouse HPs. Treatments were monitored in cells incubated under normoxic conditions and in normal or modified Krebs-Henseleit buffers (Krebs), as used previously for the Na^+^ perturbation and primary HP death studies^6–8^. To increase the physiological relevance, treatments were also performed in normal and custom-modified DMEM culture media in serum free conditions.

**Fig. 1:**
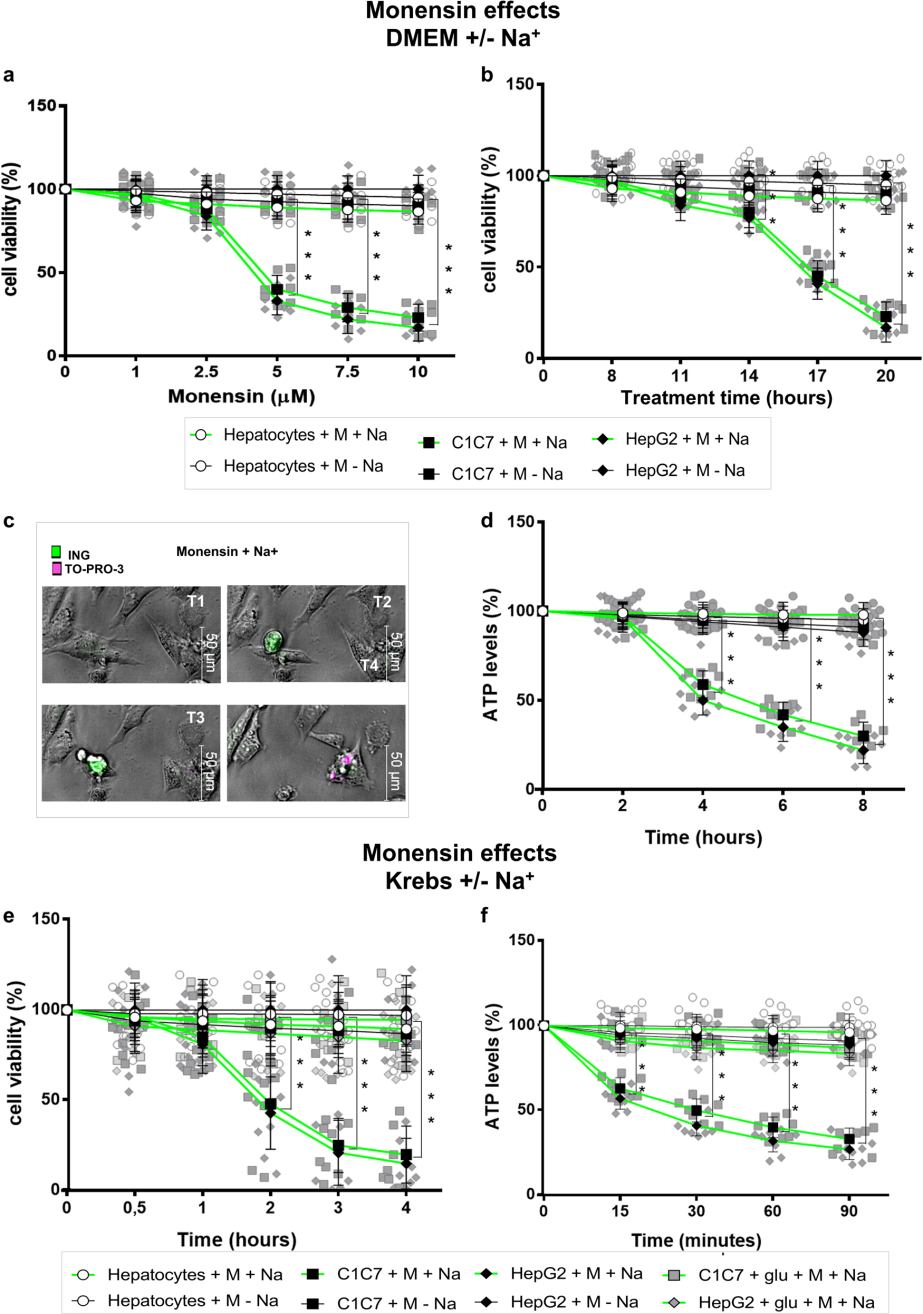
Na^+^-dependent energy depletion and killing of HCC cells treated with Monensin. **a** Dose dependent analysis of the cytotoxic effects of 20 h treatments with Monensin (M) on C1C7, HepG2 cells and hepatocytes in DMEM ± Na^+^. **b** Effects of 20 h treatments with 10 μM Monensin (M) on the viability of C1C7, HepG2 cells and hepatocytes in DMEM ± Na^+^. **c** Representative in vivo cell living images of intracellular Na+ increase (Green: ING) and appearance of cell death (Violet: TO-PRO3) of C1C7 cell exposed to Monensin 10 μM in DMEM + Na^+^. Time frame 30′: T0 0′-T1 30′-T2 60′- T3 90′ -T4 120′. **d** The ATP levels (nmol/10^6^ cells) of Hp, C1C7 and HepG2 cells upon 8 h of 10 μM Monensin (M) treatments in DMEM ± Na^+^ (levels at T0: Hp = 17.5 ±  2.1; C1C7 = 19.9 ±  1.8;HepG2 18.9 ±  1.9). **e** Viability of Hp, C1C7 and HepG2 cells with 4 h treatment of 10 μM Monensin (M) in Krebs ± Na^+^ plus or minus glucose (glu) 5 mM. **f** Intracellular ATP levels of Hp, C1C7 and HepG2 cells with 90 min treatments of 10 μM Monensin (M) in Krebs ±  Na^+^ plus or minus glucose (glu) 5 mM. Results are expressed as % of controls (*n* = 8 independent experiments). Symbols represent the average while bars represent the standard deviation. Green lines represent the presence of sodium in the extracellular medium. ***P < 0.001 with O-way Analysis of Variance (ANOVA) Bonferroni Multiple Comparisons Test. C1C7 + M + Na^+^ and HepG2 + M + Na^+^ significantly different from C1C7 + M + Na^+^ and HepG2 + M + Na^+^; Hepatocytes +M ± Na^+^ in all panels; and C1C7 + M + Na^+^ + glu and HepG2 + M + Na^+^ + glu in panel **e** (except the 2nd hour) and **f**.

Monensin killed, in a dose-dependent manner, C1C7 and HepG2 cells maintained in DMEM medium for 20 hours (h) with a maximum effect seen at a 10 μM concentration (Fig. 1a–c; Supplementary Fig. 1 and Supplementary videos 1, 2, and 5). The HCC cells incubated in Krebs buffer showed a much earlier sensitivity to Monensin, with the effects evident as early as the 2^nd^ hour of treatment and delayed by the addition of the main energy-linked component of DMEM medium, 5 mM of glucose (Fig. 1e). Accordingly, a progressive depletion of intracellular ATP preceded the beginning of HCC cell death and ATP loss was appreciable in HCC cells starting at 15 minutes (min) when incubated in Krebs (Fig. 1f), and from the 4th hour when maintained in DMEM medium (Fig. 1d). In contrast to HCC cells, Monensin treatment of HPs incubated in either Krebs or DMEM medium did not produce any significant modifications in viability and ATP content when compared to untreated HPs (Fig. 1a, b, d–f).

The incubation of HCCs in the Na^+^-free Krebs buffer^6–8^ or in the Custom-modified Na^+^-free DMEM medium, prevented the decrease of intracellular ATP as well as appearance of cell damage in Monensin-treated HCC cells (Fig. 1a, b, d–f; Supplementary Fig. 1; and Supplementary videos 3 and 4). This indicated that Monensin toxicity occurred through a Na^+^-dependent and cancer-specific mechanism that appeared to be related to energy availability.

**Fig. 2:**
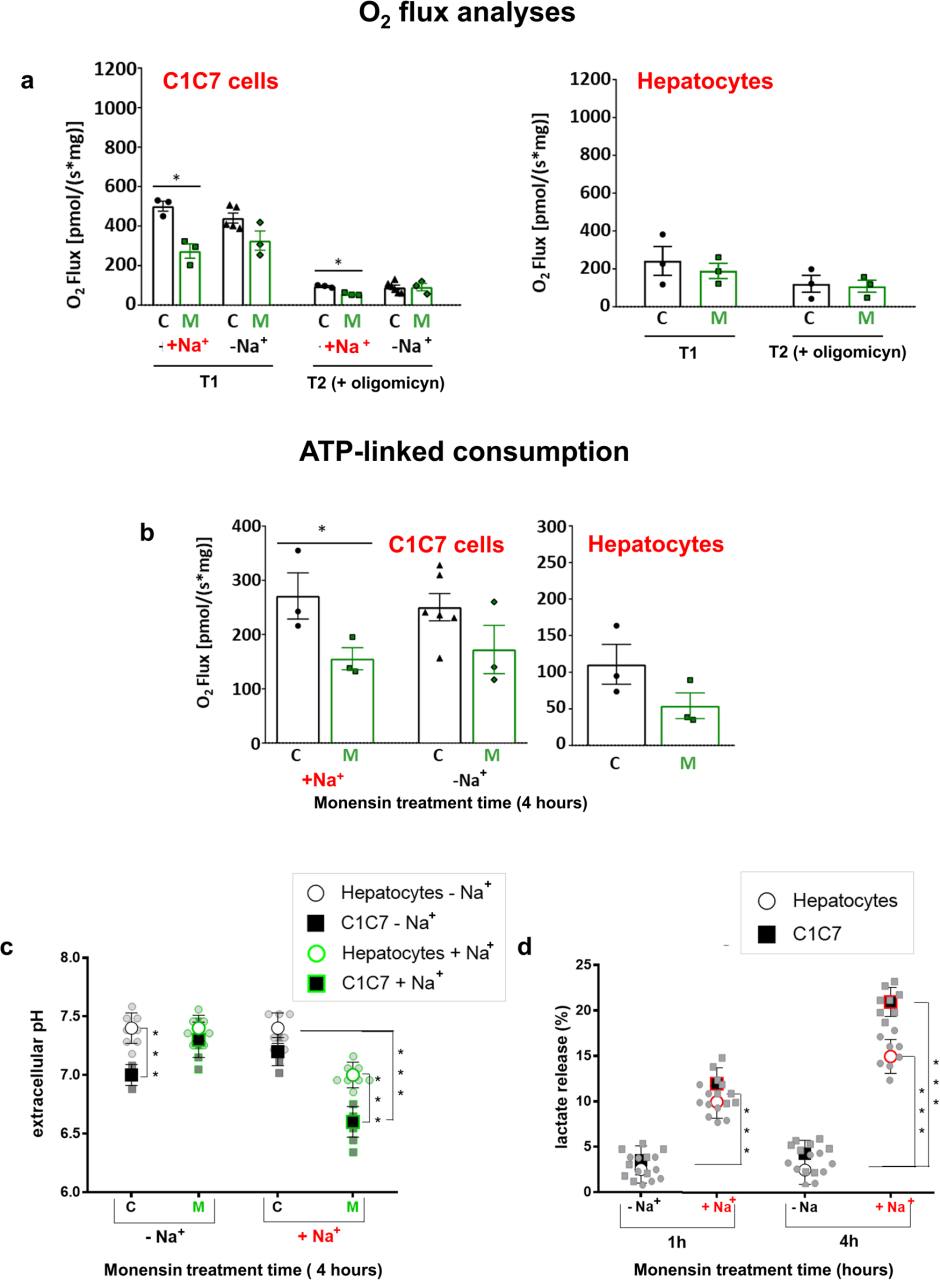
Monensin affects mitochondrial oxygen consumption in HCC cells. **a** Oxygen fluxes of C1C7 cells and hepatocytes (HPs) incubated for 4 h with (M) or without 10 μM Monensin in DMEM ± Na^+.^ prior (T1) and after (T2) the addition of the ATP synthase inhibitor oligomycin (*n* ≥ 3 independent experiments). **b** Oxygen consumption linked to ATP production (T1-T2) of C1C7 cells and hepatocytes (HPs) incubated 4 h with or without 10 μM Monensin in DMEM ± Na^+^. Data are expressed as oxygen consumption normalized to the sample protein content (*n* ≥ 3 independent experiments). **c** Extracellular pH of C1C7 cells and HPs incubated 4 h with or without 10 μM Monensin in DMEM ± Na^+^ (*n* = 8 independent experiments). **d** Lactic acid release of C1C7 cells and HPs incubated 4 h with or without 10 μM Monensin in DMEM ± Na^+^ (*n* = 8 independent experiments). Values normalized to sample protein content (**c**, **d**) and expressed as % of control values (**d**). *P < 0.05, ***P < 0.001 with unpaired T-test.

As occurs for most transformed cells, HCC cells mainly rely on aerobic glycolysis to produce energy^25^. A functional mitochondrial oxidative phosphorylation (OxPhos) may, however, act as a critical source of ATP especially under high energy-consuming conditions. Mitochondrial O_2_ consumption rate (OCR) flux analyses (Fig. 2a, b), showed that a 4 h incubation with Monensin in DMEM, significantly decreased basal and Oligomycin-sensitive oxygen consumption of HCC cells, but not for HPs (Fig. 2a, b). On the other hand, the glycolytic activity, evaluated in terms of extracellular acidification (Fig. 2c) and lactic acid release (Fig. 2d), was increased by Monensin in both HCC cells and HPs. Cells incubated in the absence of extracellular Na^+^ prevented the reduced respiratory capacity of HCC cells as well as the increased glycolytic activity of both HCC cells and hepatocytes (Fig. 2a–d), indicating that a Monensin-mediated Na^+^ influx promoted an increased glycolytic energy production but also further decreased OxPhos ATP production in HCC cells.

**Fig. 3:**
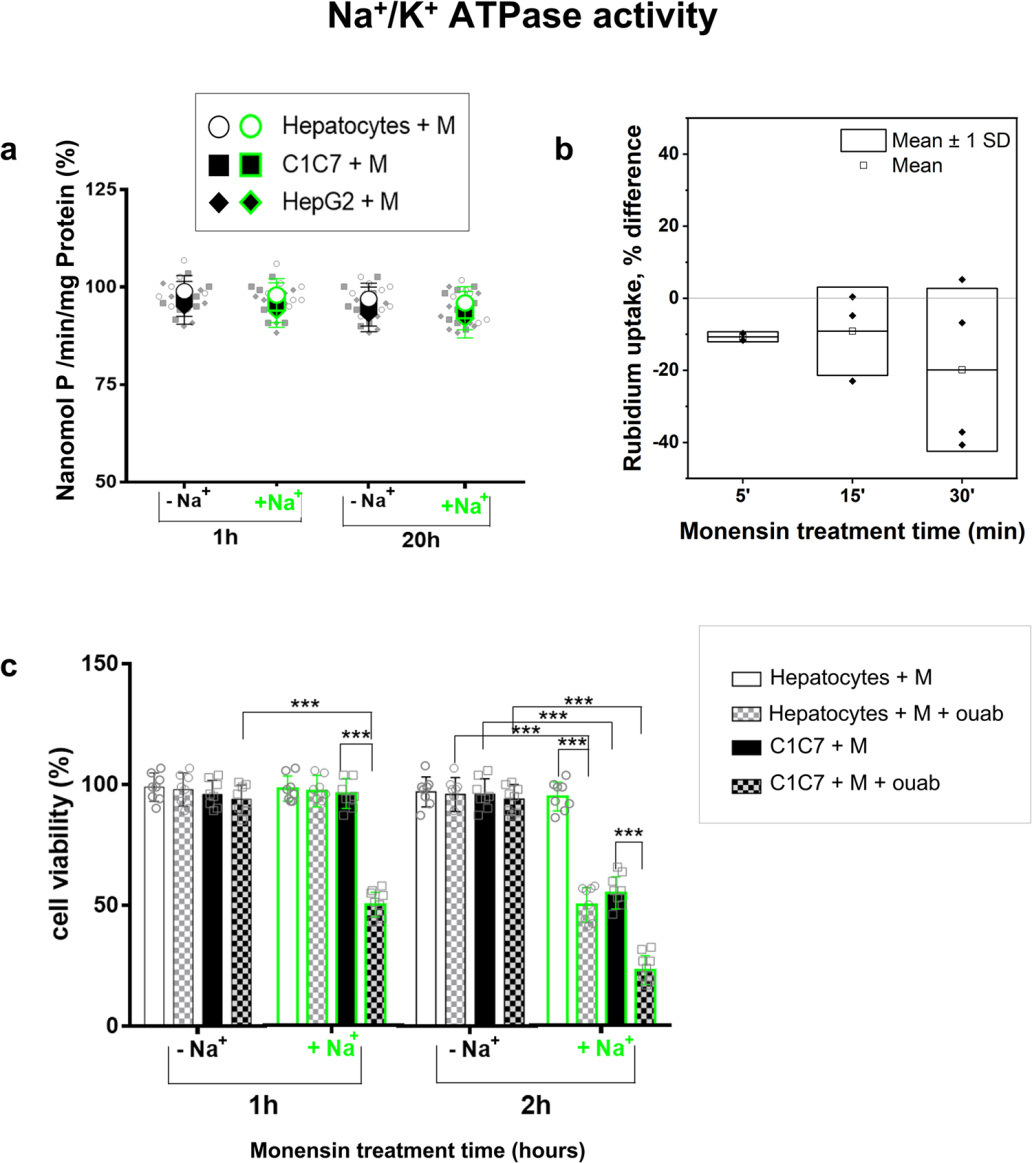
Effects of Monensin on Na^+^/K^+^ ATPase activity and of Na^+^/K^+^ ATPase inhibition. **a** Basal activity Na^+^/K^+^ ATPase in Hepatocytes (HPs), C1C7 and HEPG2 cells exposed 1 h and 20 h to Monensin 10 μM (M) in DMEM ± Na^+^, evaluated as in nmol phosphate/min/mg protein and expressed as % of control values (*n* = 4 independent experiments). **b** Functional Na^+^ /K^+^ ATPase activity expressed as % difference of the Rubidium uptake, on respect to the control, for C1C7 cells incubated in Krebs buffer with 10 μM Monensin (*n* ≥ 2 independent experiments). **c** Cell viability of C1C7 cells and of HPs incubated with 10 μM Monensin (M) plus or minus 1 mM ouabain (ouab) Krebs ±  Na^+^. Results are expressed as % of respective controls value (*n* = 8 independent experiments). Symbols or bars represent the average while error bars represent the standard deviation. ***P < 0.001 with unpaired T-test.

The highest energy-consuming enzyme is the ubiquitous plasma-membrane pump Na^+^/K^+^ ATPase, which is primarily responsible for the removal of Na^+^ from cells^26^. Monensin treatment extended up to 20 h did not affect Na^+^ /K^+^ ATPase basal activity, assessed as the capability to decompose exogenously added ATP (Fig. 3a), thus excluding a direct inhibitory effect of the ATPase pump. The functional activity of Na^+^/K^+^ ATPase, evaluated as Rb^+^ uptake capacity^17^ in the course of Monensin treatment without exogenously added ATP, highlighted an early, though not significant, reduction of the Rb^+^ uptake (Fig. 3b) that was parallel to ATP depletion (Fig. 1f) and absent in Na^+^-free conditions (Supplementary Fig. 2). This possibly suggests that the consumption of ATP to extrude sodium by the same Na^+^ /K^+^ ATPase, led to the decrease of intracellular ATP that made the same Na^+^ /K^+^ ATPase pump unable to efficiently fuel a further sodium extrusion, therefore leading to a progressive and cytotoxic sodium load. This is consistent with a critical role of the block or the reduced Na^+^ extrusion by the Na^+^ /K^+^ ATPase pump in Monensin toxicity. Accordingly, the blockage of Na^+^ /K^+^ ATPase with ouabain accelerated the appearance of Monensin-induced damage of HCC cells making primary HPs sensitive to the toxic effects of the ionophore, but only in presence of extracellular Na^+^ (Fig. 3c).

### Intracellular Na^+^ loading decreases water *k*_io_ which precedes Monensin HCC cell killing

**Fig. 4:**
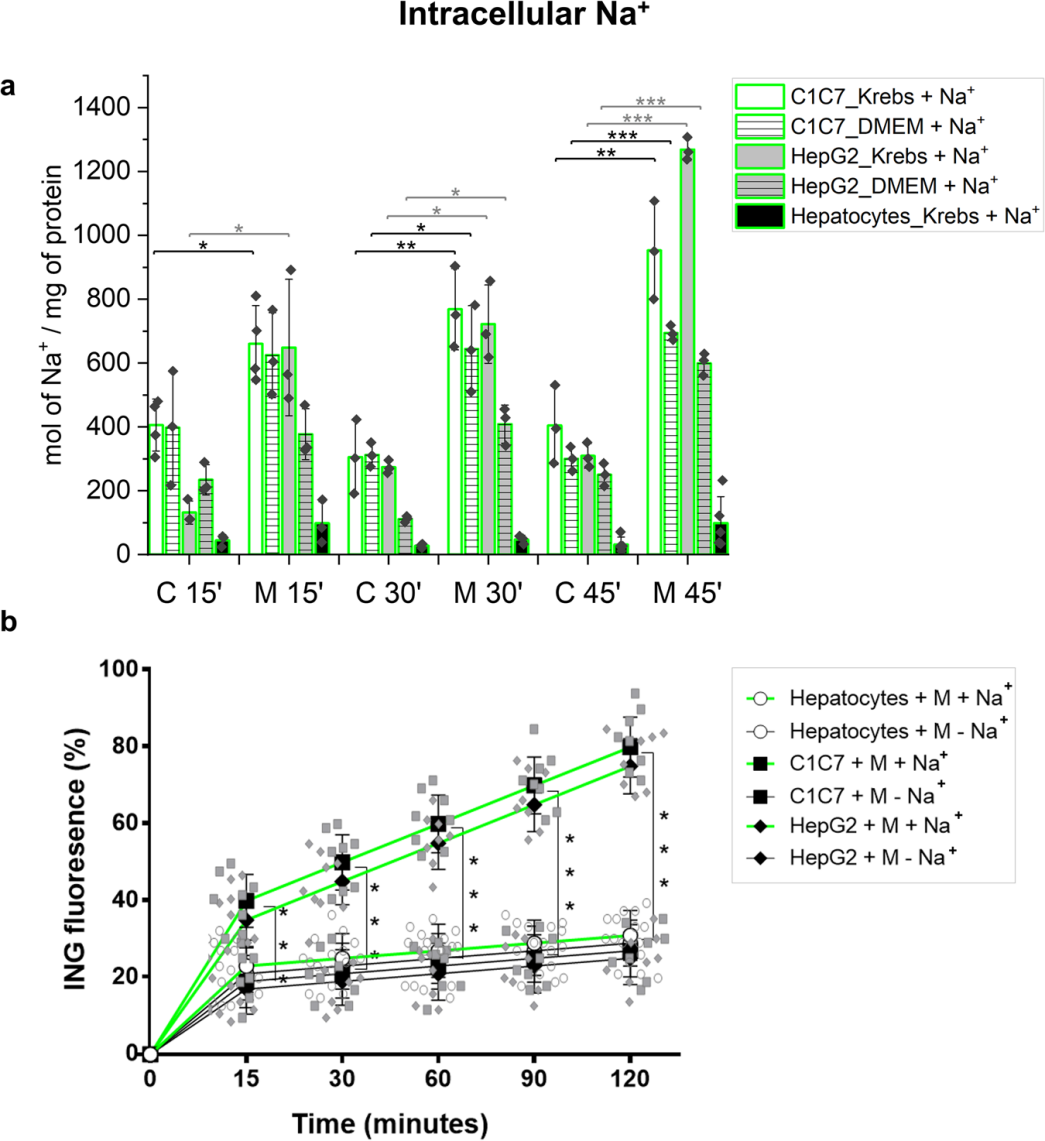
Monensin increases intracellular Na^+^ content. **a** Na^+^ uptake of C1C7 and HepG2 cells and of hepatocytes (HPs) incubated with (M) or without (C) 10 μM Monensin in Krebs ± Na^+^ or DMEM (culture medium) + Na^+^(*n* ≥ 3 independent experiments). *P < 0.05, **P < 0.01, ***P < 0.001 with the Student’s *t*-test. **b** ING fluorescence of C1C7 and HepG2 cells and of HPs incubated with (M) or without (C) 10 μM Monensin in Krebs ± Na^+^. Values are expressed as % of the respective control values: same type of cells (hepatocytes, C1C7 or HepG2 cells) incubated in absence of Monensin at the same time-point of incubation (*n* = 8 independent experiments). ***P < 0.001 with O-way Analysis of Variance (ANOVA) Bonferroni Multiple Comparisons Test. Symbols or bars represent the average while error bars represent the standard deviation.

We analyzed [Na^+^]_i_ variations by monitoring the changes of the relative fluorescence of [Na^+^]_i_ dye ION Natrium-Green AM (ING-AM) and by quantifying [Na^+^]_i_ by Inductively Coupled Plasma-Mass Spectrometry (ICP-MS). The ICP-MS analysis showed that untreated HPs had a lower content of Na^+^ (expressed as nmol of Na^+^ normalized to the mg of cellular proteins) compared to mouse and human HCC cells (Fig. 4a). This confirmed the increased intracellular sodium concentration in cancer cells compared to primary cells observed by X-ray microanalysis^9^. The different methods as well as the different cells utilized, can explain the two times lower increase reported by the earlier study^9^. Moreover, it was observed that Monensin further increased [Na^+^]_i_ of both human and murine HCC cells but did not significantly affect the Na^+^ level of HPs (Fig. 4a). The millimolar [Na^+^]_i_ was extrapolated using the experimentally determined relationship between the mg of cell proteins per cell number and an estimation of the cell volume (Supplementary Fig. 3).

**Fig. 5:**
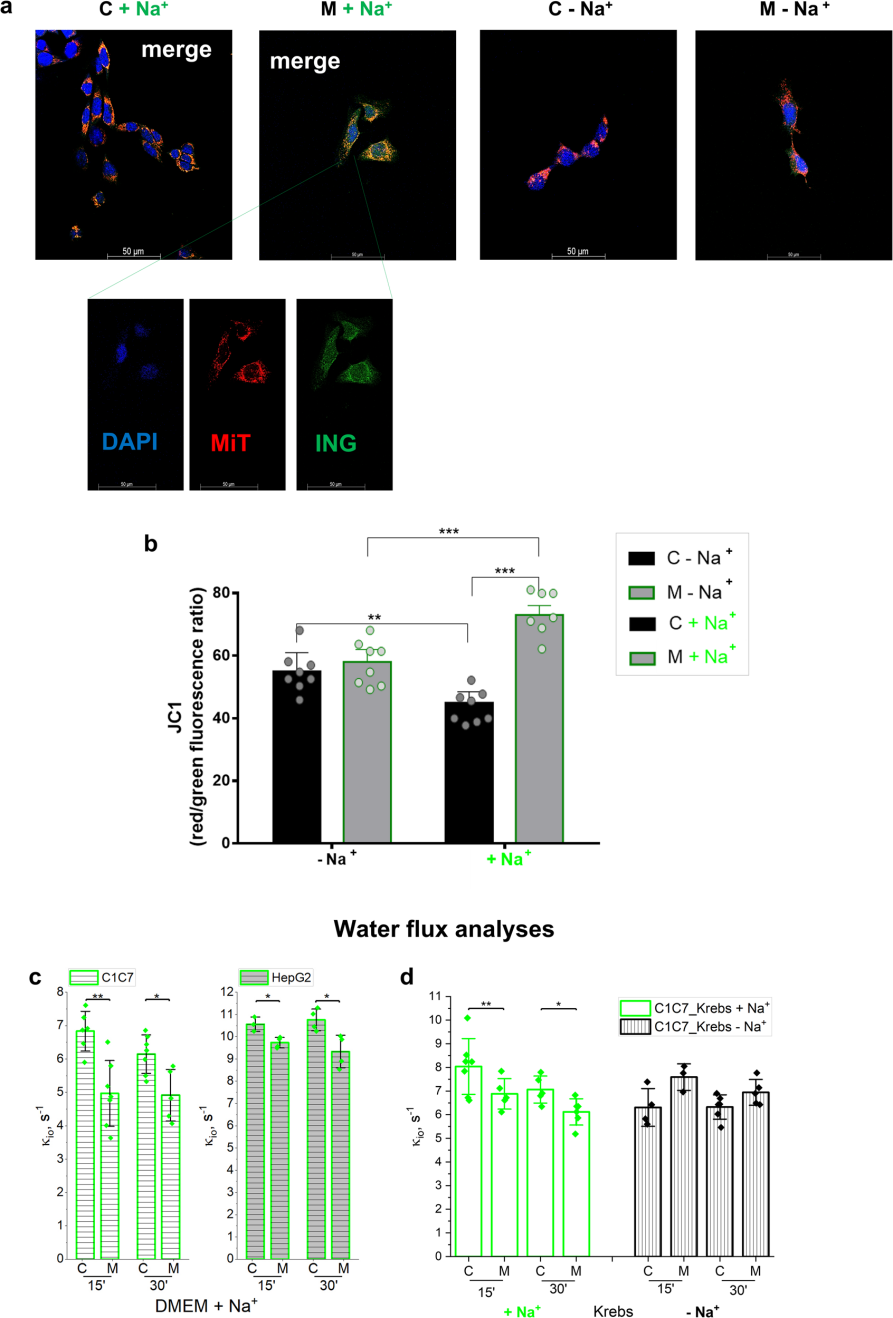
Monensin increases mitochondrial Na+ content and polarization. **a** Representative overlay confocal images of intracellular Na+ (Green: ING) and viable mitochondria (Red: MitoTrack). Cell nuclea were highlighted with DAPI (BLU). **b** Mitochondrial membrane polarization (JC1-1 red/green fluorescence) of C1C7 cell exposed (M) or not (C) 4 h to Monensin 10 μM in DMEM ± Na^+^ (*n* = 8 independent experiments).**P < 0.01, ***P < 0.001 with unpaired T-test. **c**, **d** Cellular water molecule efflux rate constants (ki_o)_ determined on HCC cells exposed (M) or not (C) to 10 μM Monensin up to 45 min in DMEM + Na^+^ (panel **c**, C1C7 on the left and HepG2 on the right) or Krebs ± Na^+^ (C1C7 cells, panel **d**) (**c**, **d:** *n* ≥ 3 independent experiments). Bars represent the average while error bars represent the standard deviation. *P < 0.05, **P < 0.01, ***P < 0.001 with the Student’s *t* test.

Fluorimetric and in vivo imaging analysis showed an increase of ING-AM fluorescence that preceded the appearance of Monensin toxicity (Figs. 4b and 1c, Supplementary Videos 1, 2, and 5). ING-AM loaded HCC cells co-stained with the Red MitoTracker dye to highlight healthy polarized mitochondria, highlighted that ING-AM co-localized with MitoTracker fluorescence upon Monensin treatment (Fig. 5a, b, Supplementary Fig. 4a, b, and Supplementary videos 6–9), likely indicating an increase of mitochondrial Na^+^ content. An increase in cation concentration in the mitochondrial matrix can be predicted to induce mitochondrial hyper-polarization. Indeed, the induction of K^+^ entry into the mitochondria has been shown to increase the mitochondrial potential^27,28^. Accordingly, the JC1 analysis showed that Monensin increased the mitochondrial potential of HCC cells (Fig. 5b). HCC cell incubation in Na^+^-free condition, inhibited the increase of intracellular and mitochondrial ING-AM fluorescence, mitochondrial hyper-polarization as well as cell permeability to the cytotoxicity detection dye TO-PRO™−3 Iodide (Fig. 5a, b, Supplementary Fig. 4c, d, and Supplementary videos 3 and 4).

As an osmotically active ion, Na^+^ is likely to drive a corresponding water influx inside cells. The role of the increased Na^+^ intracellular content and the effects of Monensin were therefore analyzed in relation to the *k*_io_ of cancerous cells by NMR relaxometric (T_1_) measurements^16–18^. HCC cells displayed a *k*_io_ in the range 6.2-11.1 s^−1^ in the culture medium that decreased significantly upon Monensin treatment (Fig. 5c, d). Interestingly, no significant *k*_io_ decrease was observed with incubation in the Na^+^-free buffer (Fig. 5d). This observation confirms the hypothesis correlating the decrease of *k*_io_ with the [Na^+^]_i_. Moreover, the observed *k*_io_ decrease may be due to the osmotically driven cell swelling.

### HCC inhibition by Monensin is associated to a cancer-selective Na^+^ increase and cell damage

**Fig. 6:**
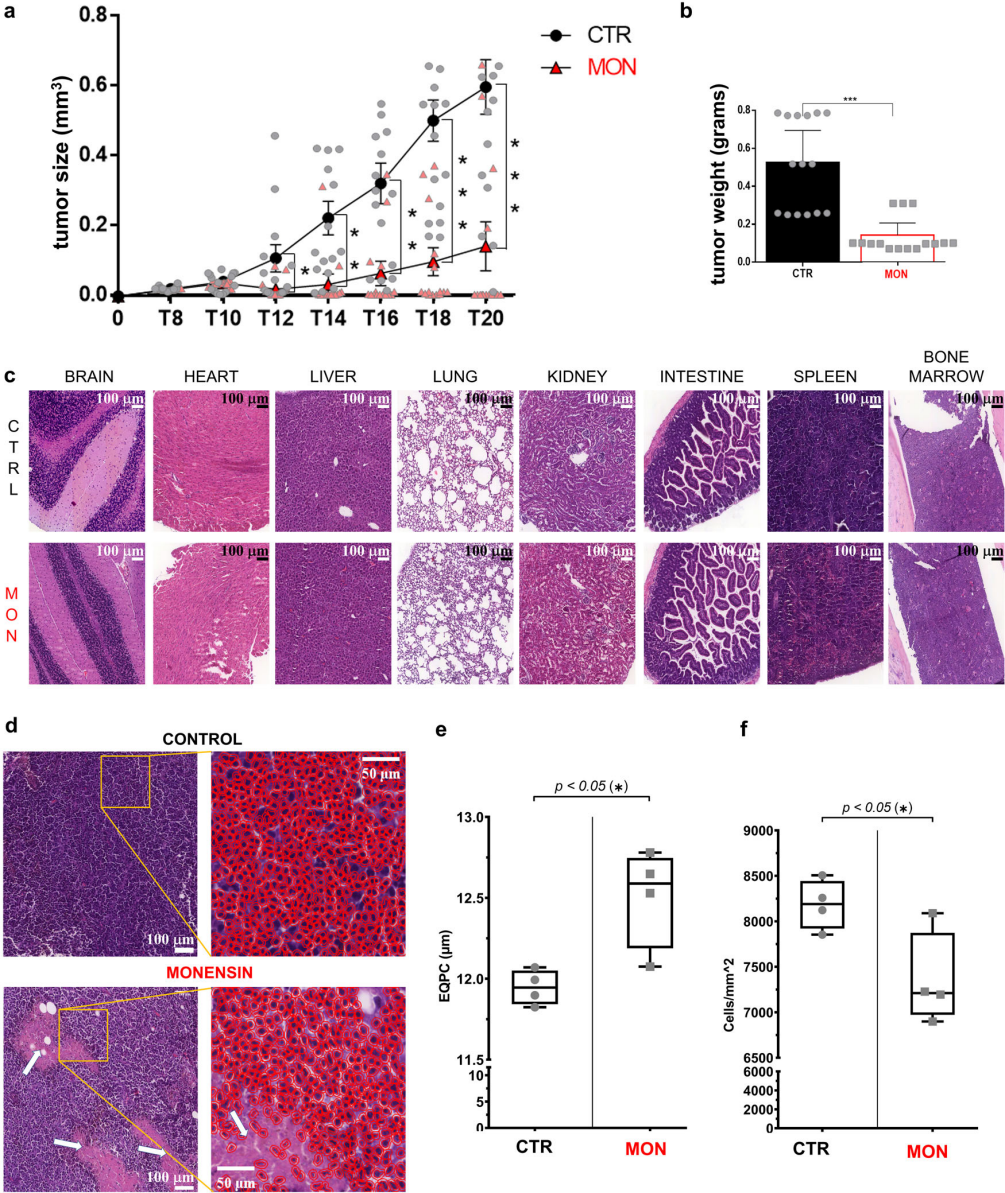
Monensin inhibits HCC development and promotes HCC necrosis without affecting vital organs. **a** Allograft growth of C1C7 cells in NSG mice i.p. treated with Monensin (8 mg/kg) (MON) or with vehicle (C) (*n* = 15 control, *n* = 15 MON treated mice). **b** Weight of extracted allograft tumors at termination (*n* = 15 control, *n* = 15 MON treated mice). **c** Representative H&E images of healthy tissues of mice i.p. treated with Monensin (8 mg/kg) or vehicle. Magnification: scale bar = 100 μm. **d** Representative H&E images of tumor allograft of mice i.p. treated with Monensin (8 mg/kg) or vehicle. Necrotic areas are indicated by white arrows. Left panels: profiles of cells evinced in red to evaluate cellular density (**e**) and size (**f**). Magnification: scale bar = 50 or 100 μm. **e** Quantification of tumor cellular density evaluated as cells/mm^2^ in vehicle or Monensin (8 mg/kg) treated mice. For each mouse 20 representative images were used (*n* = 4 control, *n* = 14 MON treated mice). **f** Quantification of cell size as EQPC (diameter of a circle of equal projection area of the cell) in vehicle or Monensin (8 mg/kg) treated mice tumors. For each mouse 20 representative images were used (*n* = 4 control, *n* = 4 MON treated mice). Hpf: high power field. Symbols or bars represent the average while error bars represent the standard deviation. *P* < 0.05, ***P < 0.001 by unpaired T test.

Anti-HCC activity of Monensin was assessed by daily i.p. treatment at 4, 8, or 16 mg/kg/day beginning at day 10 after mouse tumor (HepaC1C7 cells) allograft flank implantation (Fig. 6a and Supplementary Fig. 5a). Monensin reduced HCC tumor size starting at the 4th day of treatment with significant effects at the doses of 8 and 16 mg/kg/day (Fig. 6a and Supplementary Fig. 5a). Monensin did not affect survival, general wellness assessed by the daily monitoring, as well as body weight (Supplementary Fig. 5c) of mice. At the end point, mice were killed to evaluate cell damage, proliferation activity and Na^+^ levels of both tumor and healthy tissues.

**Fig. 7:**
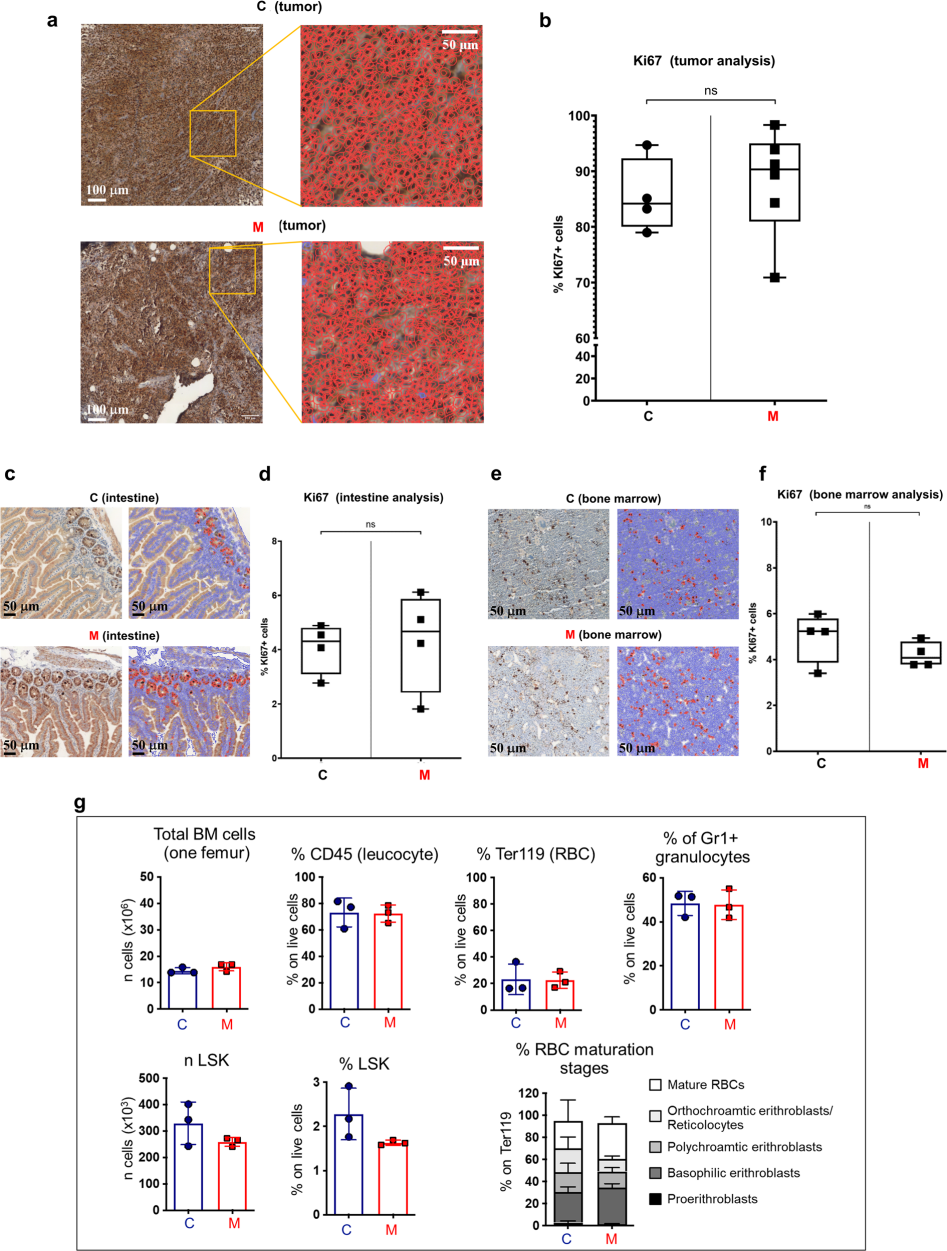
Monensin does not affect hemopoiesis and cell proliferation (ki67) of tumor, intestine and bone marrow. **a** Representative immunohistochemical staining for ki67 of tumor of mice treated with Monensin (8 mg/kg) (M) or with vehicle (C). High resolution images: profiles of the single cell outlined in red. Magnification: scale bar= 50 or 100 μm. **b** Plots show the percentage of ki67 positive cells in the tumor of mice treated with Monensin or vehicle. For each mouse 20 representative images were used. Columns represent the average while bars represent the standard deviation (*n* = 4 C, *n* = 4 M treated mice). **c** Representative immunohistochemical staining for ki67 of intestine of mice treated with Monensin or vehicle. Magnification: scale bar = 50 or 100 μm. **d** Percentage of ki67 positive cells in the intestine 4 different mice treated with Monensin or with vehicle. For each mouse 20 representative images were used. Columns represent the average while bars represent the standard deviation (*n* = 4 control, *n* = 4 M treated mice). **e** Representative immunohistochemical staining for ki67 of bone marrow of mice treated with Monensin or vehicle. Magnification: scale bar = 50 or 100 μm. **f** Percentage of ki67 positive cells in bone marrow of mice treated with Monensin or with vehicle. For each mouse 20 representative images were used. Columns represent the average while bars represent the standard deviation (*n* = 4 control, *n* = 4 M treated mice). **g** Bars plots showing the total number of cells, the percentage of CD45^+^ leukocytes, of total and relative RBC Ter119^+^ progenitors, of Gr1^+^ granulocytes; of number and percentage of LSK in BM of NSG mice injected with tumor; columns represent the average while bars represent the standard deviation; dots represent single mice (*n* = 3 control, *n* = 3 M treated mice). Statistical tests: **b**, **d**, **f** n.s (not significant) with unpaired T test; **g** not significant with unpaired nonparametric Mann Whitney *U* test; graph with % of red blood cell maturation stage, not significant with two-way ANOVA with a Sidak’s multiple comparison test.

Monensin did not provoke morphologic evidence of damage, nor did it affect the weights of vital organs such as liver, lung, spleen, kidney, heart and brain (Supplementary Fig. 5b and 6a and Fig. 6c). Monensin did not change the cell morphology and cellularity in the intestine and bone marrow (BM) (Fig. 7c, e and Supplementary Fig. 6b), or did it decrease the expression of the proliferative index ki67 (Fig. 7c–f and Supplementary Fig. 6b). Moreover, Monensin did not affect the hematopoiesis of treated NSG mice (Fig. 7g and Supplementary Fig. 8): total number of BM cells, percentage of CD45^+^ (leukocytes), Ter119^+^ (erythroid lineage), Gr1^+^ (myeloid lineage), and LSK (Lineage^-^ Sca1^+^ c-kit^+^, hematopoietic stem and progenitor) cells were equal among the two groups. Additionally, the frequency of the single erythroid progenitor populations, representing the different RBC stage of differentiation, was unaffected by the Monensin regimen. Further, Monensin treatments did not affect BM cells of NSG mice that were not implanted with the tumor (Supplementary Figs. 7 and 8).

Monensin at 8 and 16 mg/kg significantly inhibited HCC development (Fig. 6a and Supplementary Fig. 5a) and reduced HCC weight (Fig. 6b) at the end point of the study. These effects were not correlated to a reduction of ki67 (Fig. 7a, b), as observed in non-transformed proliferative tissues. In contrast, HCC inhibition by Monensin was associated with an extended tumor damage and necrosis, as demonstrated by histological observations (Fig. 6d), and which was measured by a decrease of the cellular density (Fig. 6f), as previously reported^29^. In accordance with the decreased water *k*_io_ induced by Monensin treatments in vitro (Fig. 5c, d), HCC cells of allograft in Monensin-treated mice, demonstrated a significant cell swelling in comparison to those of vehicle controls (Fig. 6e).

**Fig. 8:**
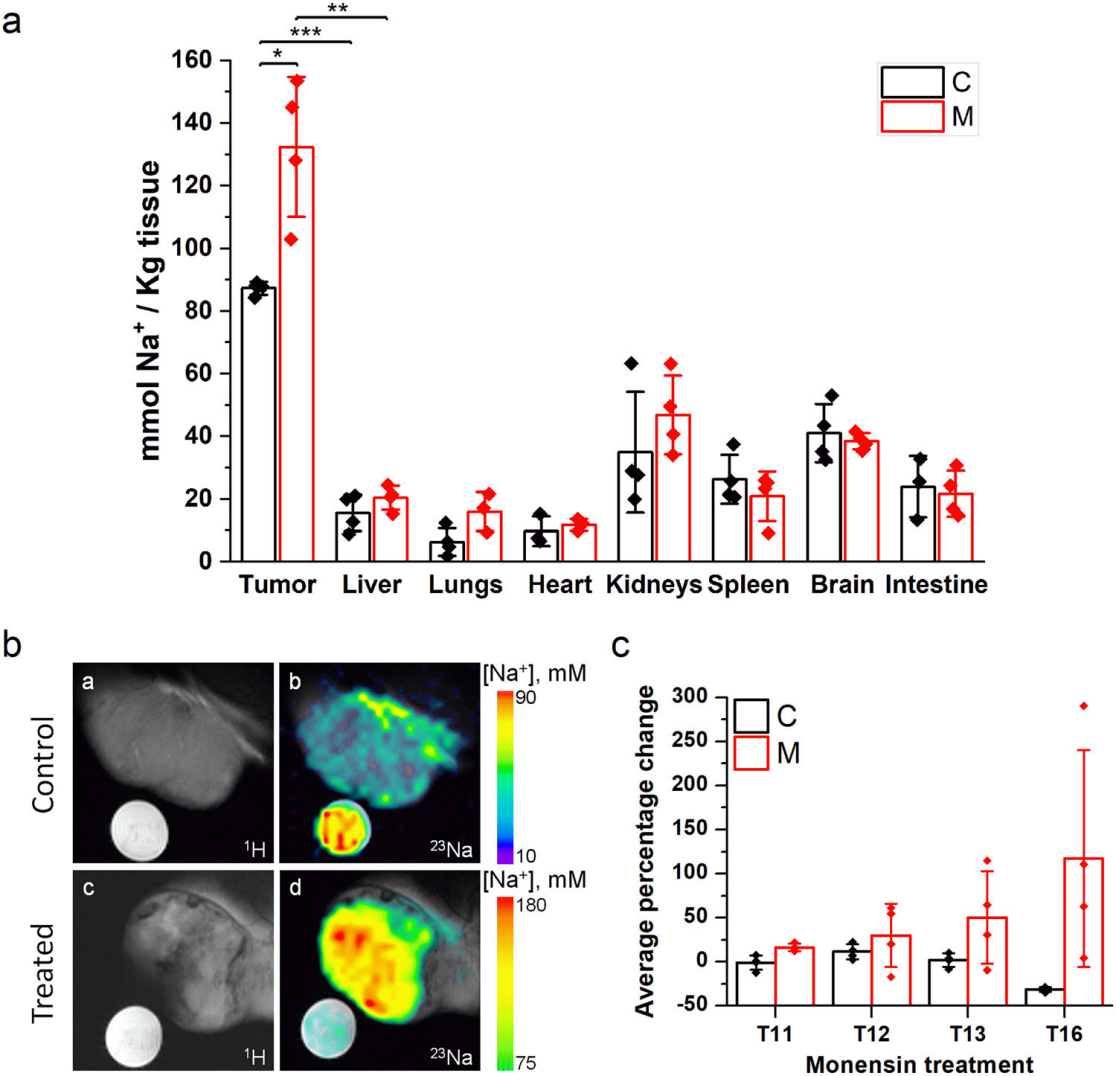
Monensin treatment increases tumor Na^+^ content and does not affect the other organs. **a** Na^+^ tissue content measured by ICP-MS in tumor and organs collected from NSG mice at the end of the treatment with Monensin (M, i.p. administration of 8 mg/Kg) or vehicle (C) (*n* ≥ 3C and *n* ≥ 3M treated mice). **b** Selected tumor MRI images of control (**a**, **b**) and treated (**c**, **d**) mouse acquired 16 days after the inoculation of C1C7 cells (T16): **a**, **c** proton T_2_-weighted images and its fusion with the correspondent ^23^Na images (**b**, **d**, the color bar indicates the millimolar tissue [Na^+^] calculated from the signal intensity of the reference sample). **c** Average percentage change calculated on the whole tumor from the ^23^Na MRI images (see text). (*n* = 4C and *n* = 3M treated mice). The time (days) after the inoculation of C1C7 cells is indicated on the x axes. Bars represent the average while error bars represent the standard deviation. *P < 0.05, **P < 0.01, ***P < 0.001 (Student’s *t* test).

Intracellular Na^+^ concentrations, determined by ICP-MS, was markedly higher in tumors than in normal livers and all the other organs investigated (Fig. 8a). Interestingly, Monensin-treated mice showed a substantial increase in Na^+^ concentration in tumor tissue with respect to the untreated controls (Fig. 8a). This increase was specific only to tumor tissues, with Na^+^ concentrations in other organs unaffected by Monensin treatment (Fig. 8a).

The results obtained with ICP-MS analysis were confirmed by ^23^Na MRI, which is able to non-invasively and directly probe Na^+^ tissue concentrations. The MRI scans were carried out on two groups of mice: a Monensin-treated HCC tumor bearing mice (images c, d of Fig. 8b) compared with an untreated control group (images a, b of Fig. 8b). Figure 8C shows the signal intensity of tumors (expressed as average percentage change) in the Monensin-treated group and control mice demonstrating, while not significant, a rising trend. Using standard tubes containing agar embedded with a 75 mM NaCl solution (Fig. 8b), it was possible to extrapolate the intra-tumor Na^+^ tissue concentration which correlated with ICP-MS measurements carried out post-mortem on the same tumor tissues (Supplementary Table 1). Further, the tumor tissue [Na^+^] of each mouse measured during the 5 days of the Monensin treatment was reported in Supplementary Fig. 9. During the treatment we observed a progressive increase of the hyperintense regions (Supplementary Fig. 10) that likely corresponded to necrotic tumor tissues.

## Discussion

This study investigated Na^+^ homeostasis as a novel target to inhibit HCC. We showed that HCC cells and allografts have an increased Na^+^ content when compared to healthy cells and tissues, and that the treatment with the Na^+^ ionophore Monensin increases further these levels without affecting the Na^+^ levels of healthy tissues. We found that a cancer-selective Na^+^ increase correlated with HCC cell killing, and a slower growth rate of HCC xenograft tumors. Herein, we report for the first time the characterization of the cellular events involved in this process. Our results show that: (i) HCC cells are, unlike healthy hepatocytes, specifically sensitive to the toxic action of the Na^+^ ionophore being energetically unable to compensate and survive the pharmacologically induced Na^+^ load; (ii) the energetic deficiency of HCC cells was associated to Na^+^-dependent alterations of the mitochondrial respiratory capacity. Several observations support these assumptions.

We found that prevention of intracellular Na^+^ increase by employing sodium-free cell incubation media prevented Monensin-induced HCC cell death, highlighting a sodium-dependence of the cancer-specific cytotoxic action of the ionophore. We observed the protective effects of the Na^+^-free media by employing both a saline buffer (i.e. Krebs Henseleit), used previously in our early study on the role of intracellular Na^+^ alterations in the final mechanisms of primary hepatocyte death following ATP depletion^6–8^, and a custom-modified sodium-free DMEM culture medium prepared specifically for the present study. These experimental conditions prevented the toxic actions of Monensin without altering intracellular pH^6^. Monensin is generally regarded as a sodium ionophore, but it may also be able to move different cations across the membrane^19,20^. The sodium concentration in extracellular medium, however, is far higher than that of any other cations, thus sodium is likely to saturate the cation-transport activity of Monensin since the relative concentration of extracellular sodium is 145 mM and that of administered Monensin is 10 μM. All experiments performed in sodium-free conditions showed a “prevention” of the cellular alterations and cytotoxicity induced by Monensin. Thus collectively they indicate that the observed toxic effects of Monensin were sodium-dependent and thus directly related to the capacity of Monensin to specifically move sodium inside of the cells.

Monensin did not affect ATP levels and the viability of primary HPs. In contrast, HCC cell killing by Monensin were preceded by a progressive ATP loss and prevention of sodium increase by cells incubated in absence of extracellular Na^+^, rescued energy depletion and appearance of cell death. Monensin did not directly inhibit basal Na^+^/K^+^ ATPase, but pharmacological blockage of the Na^+^/K^+^ pump abolished the HPs resistance to Monensin and increased its cytotoxic effects on HCC cells. This suggests that the sensitivity of HCC cells to Monensin is related to their energetic incapability to maintain Na^+^ homeostasis by efficiently fueling Na^+^ extrusion.

ATP production in HCC cells relies mainly on anaerobic glycolysis^25^. We showed that Monensin increased the glycolytic activity in both HCC cells and HPs. Such an effect was sodium-dependent and thus a likely response to the increased energetic need to extrude the excess of [Na^+^]_i_. In turn, the increased rise of acid species consequential to glycolytic activation would then further promote Na^+^ influx through the Na^+^-dependent pH buffering systems^4,5^ thus contributing to Na^+^ load.

Most cancer cells have functional mitochondria^30^ and can employ oxidative phosphorylation (OxPhos) to sustain ATP production. We showed that Monensin increased the mitochondrial Na^+^ content and decreased, by a Na^+^-dependent mechanism, both intact and ATP-linked respiration in HCC cells but not in HPs. The molecular mechanisms of such Na^+^-induced perturbations are currently unknown. Previous electron microscopic analyses of the mitochondria of L929 fibroblasts exposed to Monensin, however, highlighted the appearance of structural changes such as disorganized and expanded intra-cristal spaces^31^. In addition, the elegant characterization of the anticancer activity of Gboxin, recently showed a key pathogenic role with its accumulation in mitochondria as a positively charged molecule^29^. These observations indicate a tendency of mitochondria to act as an intracellular cation sink. Such a tendency, if overloaded (i.e by a forced intracellular Na^+^ load), may be the cause of functional and structural mitochondrial dysfunction and of loss of mitochondrial ATP production. Collectively, these observations suggest that the constitutively high [Na^+^]_i_, increased by the continuous Na^+^ influx induced by Monensin and by the glycolytic metabolism, leads to mitochondrial respiratory inhibition. This creates a vicious circle of increased energetic consumption and irreversible energetic stress that makes HCC cells unable to maintain, unlike healthy HPs, a life-compatible level of intracellular Na^+^.

Our early research with primary HPs^6–8^ indicated that in the condition of energy depletion, a Na^+^-dependent imbalance of intracellular osmotic pressure accounted for the loss of plasma membrane integrity and HP death. We have now been able to image and analyze these events, showing that Monensin progressively increases [Na^+^]_i_ prior to the appearance of HCC cell death, and demonstrating a Na^+^-dependent increase of intracellular water retention. Accordingly, we showed in vivo, increased cell sizes in HCC allografts of Monensin-treated mice and, by means of ^23^Na-MRI, the association of Na^+^ intratumoral accumulation to the appearance of hyper-intense signals likely corresponding to necrotic areas.

A crucial issue in anticancer therapy is to minimize/avoid its deleterious effects on normal tissues. We showed that Monensin displayed its cytotoxic action specifically to transformed cells and tumors without altering the general wellness of the mice, the integrity of vital organs and without showing a cytostatic effect in either normal or transformed tissues. Importantly we observed the lack of anti-proliferative activity of Monensin on intestinal mucosa and BM that are often sensitive to most anti-cancer drugs. By an accurate evaluation of the hematopoiesis process, we also showed that Monensin was devoid of effects on cellularity and stages of differentiation of hematopoietic stem cells and progenitors, including all erythropoietic cell lineages.

The lack of anti-proliferative effects of Monensin in our models of allogenic HCC was not expected, as previous investigations on anti-cancer activity of Monensin reported the inhibition of pathways related to cell proliferation^20–24^. The anticancer effects of Monensin have, however, never been previously evaluated in hepatic tumor cell lines. Additionally, our experimental settings differ from previous reports. We in fact performed Monensin treatments in serum free conditions to reproduce the in vivo environment where cells of a solid tumor may have a limited or no direct contact with serum protein^32^.

In this study we employed NGS mice to clearly dissect the direct effect of Monensin on tumor cells in absence of concomitant anti-cancer responses of immune-inflammatory reactions. It will be essential in future research to employ immune-competent models to simultaneously investigate the effects of Na^+^ ionophores on immuno-inflammation.

The high Na^+^ intracellular content is associated with alkaline intracellular pH and to a reverse pH gradient, conditions known to be permissive for cancer cell proliferation, migration, and invasion^1,2^. We show that these conditions, generally regarded as advantageous for cancer cells^1,2^, can become a specific state of cancer weakness if exacerbated by Na^+^ ionophore drugs, thus highlighting Na+ homeostasis as novel target for HCC therapy. Na+ homeostasis is regulated in both normal and cancer cells by ubiquitous systems (i.e. Na^+^/K^+^ ATPase or Na^+^ -dependent pH buffering transporters) whose main physiological role in healthy tissues make their manipulation potentially problematic in clinics. Our in vivo data demonstrates that treatment with the antibiotic Monensin is a safe alternative to disrupt Na^+^ homeostasis in HCC cells without affecting normal tissues.

The potent anticancer effects of cation ionophores are well established. In 2009 among 16,000 compounds screened, only the Monensin analog, Salinomycin, was able to kill human cancer stem cells with a 100-fold greater efficiency than the commonly used chemotherapeutic drug, paclitaxel^33^. More recently, a high content screening identified Monensin as the most powerful cytotoxic compound, amongst 2640 tested. on epithelial-to-mesenchymal transition-like cells^34^. Surprisingly, with such a potent anticancer activity, and an increasing number of “in vitro” and “in vivo” preclinical studies, Monensin has never been investigated for translational studies in humans. The potential use of Monensin in the clinic has not been discussed until now due to three reported cases of human toxicity after the accidental ingestion of Monensin^35,36^, and due to the high variability of toxic effects in different species^37^.

In this study we provide, for the first time, the proof of concept of a cancer-selective cytotoxic action of Monensin, showing the capacity of the ionophore to specifically increase the sodium content in not only HCC cells but also HCC allografts, highlighting the sensitivity of HCC cells to this pharmacologically induced Na^+^ load. These important observations emphasize the need to push forward a deeper analysis of cation ionophores, both new and previously developed Monensin analogs^38^, aiming to address lower effective doses for their safe employment as novel therapeutic agents against HCC, and potentially for cancer in general.

## Methods

### Chemicals and reagents

Collagenase (Type I), N-(2-hydroxyethyl)-piperazine-N′-(2-ethanesulfonic acid) (HEPES), fetal bovine serum (FBS), gentamycin (G), penicillin (P), streptomycin (S), glutamine (Gln), Na^+^ pyruvate (Na^+^ Pyr), oligomycin, FCCP, rotenone, antimycin, ouabain, Monensin, trypan blue, propidium iodide, (2-Hydroxypropyl)-b-cyclodextrin (HPCD), and all chemicals for buffer and reagent preparations were purchased from Sigma-Aldrich (Milan, Italy).

Eagle’s Minimum Essential medium (EMEM), Pierce BCA Protein assay kit, To-PRO^TTM^ 3-iodide and MitoTracker Red CMXRos were from Thermo Fisher Scientific (Waltham, Massachusetts, USA). Dulbecco’s modified Eagle medium culture medium (DMEM) and custom-modified Na^+^ - free DMEM (DMEM - Na^+^) without any Na^+^ components, were obtained from GIBCO (S.I.A.L group, Rome, Italy).

Ion Natrium Green and lactate assay kits were obtained from Abcam (Cambridge, UK).

JC-1 was from Adipogen Life Sciences (Liestal, Switzerland).

Pierce BCA Protein assay and assays for ATP (CellTiter) and cytotoxicity detection (CelltOX) were from Promega (Madison, Wisconsin, USA).

Gd-HPDO3A (Prohance) was kindly provided by Bracco Imaging SpA, Bracco Research Centre, (Colleretto Giacosa, TO, Italy).

### Cell isolation and culture

Hepatocytes (HP) were isolated by mice liver perfusion with collagenase and subsequent differential centrifugations at 50 g for 5 min at 4 °C, as previously reported^37^. Hepatocyte suspensions were then plated on collagen-coated culture dishes and cultured for 24 h in DMEM (w/o glutamine) containing 10% FBS, 100 U/mL P, 6 mM Glutamine (Gln), and 100 μg/ml S, prior to treatments.

Murine Hepa1c1c7 (C1C7) hepatoma cells were supplied by ECACC (Salisbury, UK) and human HepG2 cells by ATCC (Manassas, VA, USA). They were routinely cultured in DMEM w/o glutamine, supplemented with 10% FBS, 100 U/mL P, 6 mM Gln, 100 μg/ml S, and EMEM medium supplemented with 10% FBS, 100 U/mL P with 100 μg/ml S, and 1 mM Na+ pyruvate (Na^+^ Pyr), respectively. Cells were maintained in a humidified chamber at 5% CO_2_ / 95% air at 37 °C and split every 1 to 3 days. All cells tested negative for mycoplasma using the MycoAlert™ Mycoplasma Detection Kit. All materials were purchased from Lonza (Basel, Switzerland).

### Cell treatments

The HPs, C1C7 or HepG2 cells were suspended (10^6^/mL cell density) in oxygenated Krebs-Henseleit buffers with normal sodium concentration (145 mM) (Krebs + Na^+^), or without any sodium components (Krebs − Na^+^), containing 20 nmol/L HEPES (pH 7.4) and incubated at 37 C° under fluxing with a 95% O_2_/5% CO_2_ gas mixture, according to our previous studies in primary rodent hepatocytes^6–8^. Both in the Na^+^-free Krebs and the Na^+^-free DMEM, sodium is substituted by choline to maintain the normal osmolarity of the media. When indicated, cells were treated with Monensin (10 μM) or preincubated 15 min with glucose (5 mM) or ouabain (1 mM).

Alternatively, HPs, C1C7 or HepG2 cells were suspended in absence of serum in DMEM with a high glucose content (5 mM) and normal sodium content (145 mM) DMEM ( + Na^+^), or without any sodium component DMEM (-Na^+^) (GIBCO, S.I.A.L, Rome, Italy). Cells were seeded at 10^6^/mL cell density or in quadruplicates in 96-well culture plates (5000 cells/well) in the presence or in the absence of Monensin (0.5, 1, 2.5, 5, 7.5, or 10 μM). DMEM (-Na^+^) was prepared by S.I.A.L group mirroring the salt composition changes of Krebs (-Na^+^)^6–8^, and leaving unchanged all other DMEM components.

### Cell viability, ATP, Na^±^ /K^±^ ATPase activity and lactate release

Cell viability and intracellular ATP were determined using, respectively, the CellTox and Cell-Titer protocols (Promega; Madison, Wisconsin, USA). The ATP values were corrected excluding ATP released by dead cells, as determined by simultaneous CellTox and Cell-Titer analyses performed for each sample. In brief, at the indicated treatment time-points, 50 µl of cells (5000 cells/well in a 96-well format), were taken and added an opaque assay plate if in suspension, or directly processed if already seeded. A volume of 20 µl of CellTox Reagent was then added to each well and fluorescence measured at an excitation of 485 nm and emission of 520 nm. A total of 100 μl/well of CellTiter Reagent was then added to the plate containing CellTox™ Green Dye and the experimentally treated cells, following which the luminescence was measured.

The basal Na^+^/K^+^ ATPase activity and lactate extracellular release were each measured respectively with MyBiosource (San Diego, California, USA) and Abcam, (Cambridge, UK) assay kits, according to the manufacture instructions. To evaluate Na^+^ /K^+^ ATPase activity, a total of 2 × 106 cells were added to 400 µl of ice cold ATPase Assay Buffer, and incubated at 25 °C for 30 min prior to measuring the optical density at 650 nm. Viability, intracellular ATP, Na^+^/K^+^ ATPase activity and lactate release were expressed as % of an equal amount of proteins (Pierce BCA Protein assay, Waltham, Massachusetts, USA) according to the vehicle treated samples.

### Intact cell respiration and extracellular pH

Cellular respiration was determined by high-resolution respirometry using the substrate, uncoupler, inhibitor, titration protocol (SUIT), as previously described^38^. The C1C7 cells were incubated for 4 h in the presence or absence of 10 μM Monensin in DMEM ( + Na^+^) or in DMEM (-Na^+^). At the end of the treatments, cells were centrifuged at 300 g for 5 min. The supernatant pHs were immediately evaluated using a HQ440D ACH pH meter (Lainate, Mi, Italy) equipped with a microelectrode. Pellets were resuspended in mitochondrial respiration medium MiR05 (0.5 mM EGTA, 3.0 mM MgCl_2_·6H2O, 60 mM potassium lactobionate, 20 mM taurine, 10 mM KH2PO4, 20 mM HEPES, 110 mM sucrose, 1 g/L bovine serum albumin, pH 7.1) and transferred to an Oxygraph-2 K high-resolution respirometer (Oroboros Instruments, Innsbruck, Austria). Control and treated samples were assessed simultaneously. After an initial stabilization of O_2_ flux, pyruvate (5 mM) was used to sustain TCA-linked respiration. The ATP synthetase inhibitor, oligomycin (O), was added at a 5 nM final concentration, and oxygen consumption was quantified to determine the oligomycin-sensitive and -insensitive respiration. Oxygen consumption rates were calculated using the accompanying software (DatLab7, Oroboros). Rates of O_2_ consumption (flux) were normalized to total protein.

### Fluorescent cytosolic Na^+^ indicator ION NaTRIUM Green-AM loading and Na^+^ measurements

Primary mouse HPs, C1C7 or HepG2 cells were loaded with the intracellular Na^+^ -fluorescent dye ION NaTRIUM Green-AM (5 µM) in Hank’s Balanced Salt Solution (HBSS) with BSA (2%), pluronic acid (10 µM) and glucose (10 mM) for 1 h at RT. Intracellular Na^+^ variations upon Monensin treatment were routinely monitored using a Kontron SFM25 spectrofluorometer set at 525 nm excitation and at 545 nm emission wavelengths and expressed as a % of control values.

### Mitochondrial membrane potential

The C1C7 cells were plated at 3 × 10^4^ cells/well in a 48-well plate for 24 h in DMEM supplemented with 10% FBS. For treatments, cells were washed with fresh DMEM with or w/o Na^+^ and then exposed for 4 h to 10 µM Monensin or DMSO in DMEM ( + Na^+^) or in DMEM (-Na^+^) in absence of FBS. After treatment, the cells were incubated in presence of 10 µg/mL JC-1 added to the culture medium for 15 min at 37 °C in the dark. Images were acquired with a fluorescence microscope (EVOS™ XL Core Imaging System, Thermo Fisher Scientific), and analyzed using ImageJ software v 1.53e, calculating the red/green fluorescence ratio (fluorescence: measured at an absorbance of 490/525 nm and 540/525 nm).

### In vivo Cell imaging analysis of ING/ToPRO3 fluorescence

The C1C7 cells were seeded in a 96-well plate for 24 h in DMEM supplemented with 10% FBS, then loaded with the intracellular Na^+^ -fluorescent dye ION NaTRIUM Green-AM. After washing with DMEM supplemented with 10% FBS, cells were treated with Monensin (10 µM) in presence of the cell-impermeant TO-PRO™−3 Iodide dye (1 µM) to selectively stain nuclei of dead cells. The cells were incubated in controlled atmosphere (5% CO_2_; 37 °C) for 20 h in DMEM ( + Na^+^) or in DMEM (-Na^+^) in the cage incubator of the THUNDER Imager 3D Live Cell **(**Leica Microsystems; Germany). The Na^+^ intracellular variations and appearance of cell death were recorded every 30 min by employing a semi-automated recording/analysis protocol of Thunder live-cell imager’ platform. Negative controls were performed using samples loaded with the singular dyes or without dyes. Changes of fluorescence were analyzed using ImageJ software v 1.53e.

### THUNDER settings

The visualization of the sodium and the TO-PRO-3 dye in the C1C7 cells in a monolayer culture, was achieved using a widefield microscope (Leica DMI8, Thunder 3D Live Cell Imaging System) equipped with a Spectra X light engine or LED8 multi-LED light source (Leica Microsystems; Germany) and a 4.2 MP DFC9000GT sCMOS camera (Leica Microsystems; Germany). Brightfield and fluorescent multi-channel images were acquired using a 20X (N PLAN L 20x/0.35 DR; Leica Microsystems, Germany) objective.

Different combinations of LED excitation light and fluorescent filter cubes were used to visualize the fluorescent probes: TO-PRO-3 (640 nm excitation, DFT51011 filter set), ION NaTRIUM Green-AM (500/20 nm excitation, YFP filter cube). Identical parameters were applied for all quantified images. All data were saved as LIF files until further processing.

### DAPI/Mitotracker/ING fluorescence and Mitotracker/ING colocalization

The C1C7 cells were seeded on coverslips for 24 h, then loaded with the intracellular Na^+^ -fluorescent dye ION NaTRIUM Green-AM, as previously described. After a wash with 1X PBS, the coverslips with the attached cells were placed in DMEM with or w/o Na^+^ Monensin (5 µM) for 4 h. After the end of the treatment, the cells were stained with 100 nM of the mitochondrial potential-sensitive fluorescent dye MitoTracker Red [45 min, controlled atmosphere (5% CO_2_; 37 °C)]. Then the cells were stained with 0.5 mg/mL of the fluorescent dye 4,6-diamidino-2-phenylindole-dihydrochloride (DAPI, Sigma-Aldrich) for 5 min at RT to highlight the cell nuclei. Cells were then washed with 1X PBS and fixed with 4% paraformaldehyde. Negative controls were performed using samples without dyes. The sequential image recording was done in a unidirectional mode with a Leica SP8 confocal microscope system (Leica Microsystems; Germany). The DAPI was excited with a 405 nm laser and emission collected from 410 to 509 nm;ING was excited with a 514 nm laser and emission collected from 519 to 589 nm;Mitotracker was excited with a 633 nm laser and emission collected from 638 to 776 nm. Images were acquired using a Leica HC PL APO CS2 63×/1.40 OIL objective at 999.66 milli absorption unit (mAU).

### Confocal settings

Samples were imaged using a Leica SP8 laser scanning confocal microscope, equipped with an Argon laser, and a Leica HC PL APO CS2 63×/1.40 OIL lens. Pinhole size was set to 1 AU. Samples were illuminated with 405, 514 and 633 nm lasers at 0.75, 1.50 and 3.0 mW, respectively. 1024 × 1024-pixel images were acquired at 600 Hz (scan speed) using HyDs and PMTs detectors. DAPI acquisition parameters were: 410-509 nm (emission wavelength collection range), 0 (gain) and 0 (offset). ION NaTRIUM Green-AM acquisition parameters were: 519-589 nm (emission wavelength collection range), 0 (gain) and 0 (offset), MitoTracker Red acquisition parameters were: 638-776 nm (emission wavelength collection range), 0 (gain) and 0 (offset). Line average of 1 was applied to all the channels. All data were saved as LIF files until further processing.

### In vitro determination of cellular water molecule efflux rate constant (*k*_io_) by relaxometric procedure after Monensin treatment

The determination of *k*_io_ was performed as previously described by Ruggiero et al.^16–18^. The C1C7 and HepG2 cells were seeded in a 175 cm^2^ flask at the density of 10-12 ×10^6^ cells/flask. The cells were detached with 0.05% trypsin and 0.02% EDTA.

The recovered pellet was resuspended in the Krebs buffer, or Krebs w/o Na^+^ buffer or in the culture medium, in the presence of the vehicle (DMSO) or 10 µM Monensin (control and treated sample, respectively). In the case of re-suspension in buffer, a previous washing step in the same buffer was performed. During the treatment times (15, 30, or 45 minutes), the cells were maintained at 37 °C and 5% CO_2_.

After treatment, the cells were centrifuged and resuspended in 1 mL of the previous solution to which was added 10 mM Gd-HPDO3A (Prohance). The relaxometric measurements were carried out within 15 min, during which cell suspensions were transferred to 5 mm NMR tubes and centrifuged for 5 min at 0.1 rcf (4 °C). Water proton *T*_1_ of the cellular pellets was measured at 0.5 T and 25 °C on a Stelar SPINMASTER spectrometer (Stelar, Mede, PV, Italy) using the inversion-recovery (IR) pulse sequence with 32 τ increments (logarithmically distributed in the 0.01–4 s range) and 2 scans. A biexponential recovery of the magnetization was observed. The *k*_io_ was determined by analyzing the IR data in terms of the 2SX model [14-16, 18) using the equation described in the following paragraph.

### The 2SX model for relaxation data analysis

In a cell suspension, water molecules distributed in the extracellular (ex) space and in the intracellular (in) cytoplasm, and exchange between the two compartments with a water exchange rate efflux rate constant *k*_io_ (=τ_in_^−1^, the average intracellular residence time reciprocal) and influx rate constant *k*_oi_ (=τ_ex_^−1^, the average extracellular residence time reciprocal), respectively. These parameters are related by the equilibrium mass balance *k*_io_ ∙ *V*_i_= *k*_oi_∙*V*_ex_, where *V*_in_ and *V*_ex_ (=1−*V*_in_) are the intracellular and extracellular water mole fractions, respectively.

From the relaxometric point of view, each environment is characterized by its own proton longitudinal relaxation rate constant, the longitudinal extracellular (*R*_1ex_) and intracellular (*R*_1in_) relaxation rate constants.

The addition of a sufficient concentration of the extracellular contrast agent (CA) Gd-HPDO3A to the cell suspension allowed the condition *k*_io_ + *k*_oi_ ~ |*R*_1ex_-*R*_1in_| to be satisfied. As such, the water exchange modulates the observed relaxation behavior, as described by the Bloch-McConnell equations^14,15,39,40^. The recovery of longitudinal magnetization after inversion is not monoexponential and is expressed as:

1 $${M}_{{{{{{\mathrm{z}}}}}}}={M}_{0}\cdot \left\{1-2\left[\left(1-{a}_{s}\right)\cdot \exp \left(-t\cdot {R}_{1{{{{{\mathrm{L}}}}}}}\right)+\,{a}_{{{{{{\mathrm{s}}}}}}}\cdot \exp \left(-t\cdot {R}_{1{{{{{\mathrm{s}}}}}}}\right)\right]\right\}$$

where *M*_z_ is the instantaneous magnetization, *M*_0_ is its Boltzmann equilibrium value, *a*_L_ and *R*_1L_ are the fraction and rate constant for the apparent component with the longer *T*_1_ (*T*_1L_ = *R*_1L_^–1^), a_S_ and *R*_1S_ are the fraction and rate constant for the apparent component with the shorter *T*_1_ (T_1S_ = *R*_1S_^–1^), and *t* is the running time for recovery by relaxation. Because *a*_L_ and *a*_S_ are related (*a*_S _+ *a*_L_ = 1), there are only three independent parameters: *R*_1L_, *R*_1S_, and *a*_S_ (or *a*_L_), expressed as:

2 $${R}_{1{{{{{{\mathrm{ex}}}}}}}}={r}_{1}\left[{{{{{{\mathrm{CA}}}}}}}\right]+\,{R}_{1{{{{{{\mathrm{ex}}}}}}}}^{0}$$

$${R}_{1{{{{{\mathrm{L}}}}}}}=	 \frac{1}{2}\left({R}_{1{{{{{{\mathrm{in}}}}}}}}+\,{R}_{1{{{{{{\mathrm{ex}}}}}}}}+\,{\tau }_{{{{{{{\mathrm{in}}}}}}}}^{-1}+\,\frac{{V}_{{{{{{{\mathrm{in}}}}}}}}}{{\tau }_{{{{{{{\mathrm{in}}}}}}}}\left(1-{V}_{{{{{{{\mathrm{in}}}}}}}}\right)}\right)\\ 	-\frac{1}{2}{\left[{\left({R}_{1{{{{{{\mathrm{in}}}}}}}}-{R}_{1{{{{{{\mathrm{ex}}}}}}}}+{\tau }_{{{{{{{\mathrm{in}}}}}}}}^{-1}-\frac{{V}_{{{{{{{\mathrm{in}}}}}}}}}{{\tau }_{{{{{{{\mathrm{in}}}}}}}}\left(1-{V}_{{{{{{{\mathrm{in}}}}}}}}\right)}\right)}^{2}+\frac{4{V}_{{{{{{{\mathrm{in}}}}}}}}}{{\tau }_{{{{{{{\mathrm{in}}}}}}}}^{2}\left(1-{V}_{{{{{{{\mathrm{in}}}}}}}}\right)}\right]}^{\frac{1}{2}}$$

$${R}_{1{{{{{\mathrm{s}}}}}}}=	 \frac{1}{2}\left({R}_{1{{{{{{\mathrm{in}}}}}}}}{+R}_{1{{{{{{\mathrm{ex}}}}}}}}+\,{\tau }_{{{{{{{\mathrm{in}}}}}}}}^{-1}+\,\frac{{V}_{{{{{{{\mathrm{in}}}}}}}}}{{\tau }_{{{{{{{\mathrm{in}}}}}}}}\left(1-{V}_{{{{{{{\mathrm{in}}}}}}}}\right)}\right)\\ 	+\,\frac{1}{2}{\left[{\left({R}_{1{{{{{{\mathrm{in}}}}}}}}{-R}_{1{{{{{{\mathrm{ex}}}}}}}}+{\tau }_{{{{{{{\mathrm{in}}}}}}}}^{-1}-\frac{{V}_{{{{{{{\mathrm{in}}}}}}}}}{{\tau }_{{{{{{{\mathrm{in}}}}}}}}\left(1-{V}_{{{{{{{\mathrm{in}}}}}}}}\right)}\right)}^{2}+\frac{4{V}_{{{{{{{\mathrm{in}}}}}}}}}{{\tau }_{{{{{{{\mathrm{in}}}}}}}}^{2}\left(1-{V}_{{{{{{{\mathrm{in}}}}}}}}\right)}\right]}^{\frac{1}{2}}$$

3 $${a}_{{{{{{\mathrm{s}}}}}}}=	\frac{1}{2}\left(1-\frac{\left({R}_{1{{{{{{\mathrm{in}}}}}}}}-{R}_{1{{{{{{\mathrm{ex}}}}}}}}\right)\left(1-2{V}_{{{{{{{\mathrm{in}}}}}}}}\right)+\,{\tau }_{{{{{{{\mathrm{in}}}}}}}}^{-1}+\,\frac{{V}_{{{{{{{\mathrm{in}}}}}}}}}{{\tau }_{{{{{{{\mathrm{in}}}}}}}}\left(1-{p}_{{{{{{{\mathrm{in}}}}}}}}\right)}}{{\left[{\left({R}_{1{{{{{{\mathrm{in}}}}}}}}{-R}_{1{{{{{{\mathrm{ex}}}}}}}}+{\tau }_{{{{{{{\mathrm{in}}}}}}}}^{-1}-\frac{{V}_{{{{{{{\mathrm{in}}}}}}}}}{{\tau }_{{{{{{{\mathrm{in}}}}}}}}\left(1-{V}_{{{{{{{\mathrm{in}}}}}}}}\right)}\right)}^{2}+\frac{4{V}_{{{{{{{\mathrm{in}}}}}}}}}{{\tau }_{{{{{{{\mathrm{in}}}}}}}}^{2}\left(1-{V}_{{{{{{{\mathrm{in}}}}}}}}\right)}\right]}^{\frac{1}{2}}}\right)$$

where *R*_1ex_^0^ is the extracellular relaxation rate with no CA, [CA] is the millimolar concentration of the CA in the extracellular space, and *r*_1_ is its relaxivity value.

The Gd concentration in the pellet was determined by a relaxometric method, after overnight mineralization of the sample in 6M HCl at 120°. The treatment caused the matrix digestion and the release of the metal as free aquo-ion. The R1 of this solution was measured at 21 MHz and 25 °C on a Stelar SpinMaster Relaxometer (Mede, Pavia, Italy) and the Gd concentration was calculated using a tailored calibration curve obtained from standard solutions of GdCl_3_^41–43^.

Since the metal complexes distribute only in *V*_ex_, the latter was determined following the relationship:

4 $${V}_{{{{{{\rm{ex}}}}}}}={n}^{{{{{{\rm{Gd}}}}}}}({{{{{\rm{mol}}}}}})/({M}^{{{{{{\rm{Gd}}}}}}}({{{{{\rm{mol}}}}}}/{{{{{\rm{l}}}}}})\ast {{{{{{\rm{Vol}}}}}}}_{{{{{{\rm{p}}}}}}}({{{{{\rm{l}}}}}}))$$

were *n*^Gd^ is the number of Gd moles determined by relaxometry; and *M*^Gd^ is the concentration of the Gd-HPDO3A solution added to the cells, and Vol_p_ is the volume of the wet cellular pellets.

### Determination of Na^+^ and rubidium uptake by cell samples

For the uptake experiments, the cells were detached as previously reported for the relaxometric experiment to obtain a pellet of approximately 2 million of cells. For Na^+^ uptake, cells were suspended in 1 ml of buffer (Krebs with or without Na^+^) or culture medium and incubated (37 °C, 5% CO_2_) in the presence of the vehicle (DMSO) or 10 μM Monensin for 15, 30 or 45 min. The cells were then washed three times with 15 mL of cold Krebs w/o Na^+^ buffer and reprecipitated by centrifugation (0.2 rcf, 4 °C). Cells were recovered by adding 200 µL of bi-distilled water. In the case of the rubidium uptake experiment, the protocol was the same, except for the presence of 0.22 mM RbCl in the 1 mL of suspension solution and for the incubation times (5, 15, and 30 min).

After sonication, the sample protein concentration was assessed by Bradford assay. The cell samples were then digested after the addition of the same amount of HNO3 (70%), using a microwave (ETHOS UP Milestone, Bergamo, Italy) heating program which consisted of a ramp up to 150 °C in 8 min, followed by 6 min at 150 °C. After mineralization, 3 mL of ultrapure water were added to the samples for ICP-MS analysis. The quantification of Na^+^ and Rb^+^ was performed by an ICP-MS (Element-2; Thermo-Finnigan, Rodano (MI), Italy) analysis. The calibration curves were obtained using Na and Rb standard solutions (Sigma-Aldrich) in the range 0.7–0.2 μg/mL and 0.2–0.005 μg/mL for Na^+^ and Rb^+^, respectively. The results were expressed as nmol of Na^+^ per mg of proteins and for Rb as percentage difference of Rb uptake with respect to the control as follows:

[(nmol of Rb^+^ /mg of protein)_t_–(nmol of Rb^+^ /mg of protein)_c_]/(nmol of Rb^+^ /mg of protein)_c _× 100 where t and c indicated the treated and control sample, respectively.

### In vivo experiments

#### Animals

Six-to-eight-week-old adult male NOD.Cg-Prkdc^scid^Il2rg^tm1Wjl^/SzJ (Jackson stock No 005557) (NSG) mice were maintained under pathogen-free conditions in the animal facility of Università del Piemonte Orientale, Department of Health Sciences, and treated in accordance with the University Ethical Committee and European guidelines (Experimental protocol authorization n. 851/2020-PR, released on 19/08/2020 from Italian Ministry of Health for protocol n. DB064.60). The mice were injected subcutaneously, in the back, with C1C7 cells (1 × 10^6^ in 100 μL of NaCl 0.9%/mouse) and the tumor growth was monitored daily. When tumor size was ~80 mm^3^ (about 10 days after the tumor cell injection), mice were treated daily with intraperitoneal injection of Monensin, 4–8–16 mg/kg dissolved in DMSO/HPCD 10% in NaCl 0.9% (1:9, v/v), or with vehicle alone (DMSO/HPCD 10% in NaCl 0.9% (1:9, v/v). 4 animals for groups were employed. Palpable tumors were used for caliper measurement in two dimensions to estimate tumor volume according to the equation *V* = (L × W x W)/2. The treatment was carried out 10 times and the mice were sacrificed 2 h after the last administration or when they displayed sufferance. At the end of the experiment, before organ harvesting, mice were perfused with Krebs w/o Na^+^ to eliminate possible contaminations arising from the intravascular Na^+^.

#### Histology and immunohistochemistry

Mouse tissues were collected for fixation in 10% neutral buffered formalin and included in paraffin. Bone marrow (BM) samples were decalcified for 8 h using an EDTA-based decalcifying solution, washed in flowing water (1 h), and subsequently processed and embedded in paraffin. Four-micron-thick sections were deparaffinized, rehydrated, and stained with hematoxylin and eosin for histopathologic analyses or with anti-Ki-67 (1:250, Ventana ® Medical Systems, Roche, Monza, Italy) to evaluate cell proliferation by using an automated immunostainer (Ventana, Roche, Monza, Italy). Slide images were acquired with the slide scanner Pannoramic MIDI 1.17; Objective tipe: Plan-Apochromat; Magnification: 20X; Camera: Zeiss ICc1. The Ki67 and cell-detection, to quantify cell density (evaluated as cells/mm^2^) and size (evaluated as EQPC: diameter of a circle of equal projection area of the cell), was conducted using QuPath’s built-in “Positive cell detection”^42,44^ Software version: QuPath-0.3.2.

#### Organ collection for flow cytometry analysis

One femur per mouse was collected and maintained in cold RPMI 1640 medium supplemented of 5% FBS. The BM cells were harvested by flushing the bones with a 26G needle and passed through a 40 μm cell filter to obtain single cell suspension. Cell count was performed by Burker chamber using trypan blue to exclude dead cells. After counting, the cells were resuspended in FACS Buffer (PBS, 2% FBS, 2 mM EDTA). Samples were stained with fluorochrome-labeled monoclonal antibodies (Table. 1) against mouse markers. A master mix of antibodies was made for each staining in FACS buffer, where the cells were washed and resuspended in master mix and incubated for 15 min at 4 °C. Samples were acquired on the Attune NxT Acoustic Focusing Cytometer (Thermo Fisher Scientific) and analyses were performed by FlowJo v10 software (BD Biosciences).

**Table. 1:** List of flow cytometry antibodies used for the murine BM cell analysis.

Antibody	Reactivity	Fluorochrome	Clone	Manufacturer	Incubation condition	Antibody dilutions
CD11b	Mouse	PE	M1/70	ThermoFisher	15 min at 4 °C	1:200
CD3	Mouse	PE	145-2C11	ThermoFisher	15 min at 4 °C	1:100
Ter119	Mouse	PE	TER-119	ThermoFisher	15 min at 4 °C	1:150
Gr1 (Ly-6G/Ly-6C)	Mouse	PE-Cy5.5	RB6-8C5	ThermoFisher	15 min at 4 °C	1:300
Gr1 (Ly-6G/Ly-6C)	Mouse	PE	RB6-8C5	ThermoFisher	15 min at 4 °C	1:300
Sca1	Mouse	PE-Cy7	D7	ThermoFisher	15 min at 4 °C	1:60
CD44	Mouse	APC	IM7	ThermoFisher	15 min at 4 °C	1:625
CD117 (c-kit)	Mouse	APC	2B8	ThermoFisher	15 min at 4 °C	1:100
B220	Mouse	PE	RA3-6B2	ThermoFisher	15 min at 4 °C	1:100
CD45	Mouse	APC-eFluor780	30-F11	ThermoFisher	15 min at 4 °C	1:100

#### Determination of Na^+^ content in tissues

At the end of Monensin treatment, implanted mice were perfused with Krebs (- Na^+^) to eliminate possible contaminations arising from the intravascular Na^+^ and sacrificed. Their organs were collected, weighed and digest in concentrated HNO_3_ (70%). Aliquots of the digested samples (0.25 mL), were mineralized by heating under microwave (ETHOS UP Milestone, Bergamo, Italy) following a program which consisted of a ramp to 160 °C in 8 min, followed by 6 min at 160 °C. After mineralization, 4 mL of ultrapure water were added to the sample volumes. The recovered samples were appropriately diluted for ICP-MS (Element-2; Thermo-Finnigan, Rodano (MI), Italy) analysis.

#### MRI acquisition

MRI images were acquired on a 7T Bruker Biospin Pharmascan 70/16 scanner equipped with a ^1^H/^23^Na transmit-receive surface coil (20 mm diameter) (Bruker, Magnetic Resonance Imaging, Milano, Italy) in the presence of a reference with known Na^+^ concentration (1% Agarose gel in Na^+^ Phosphate buffer, [Na^+^] = 75 mM).

The surface coil was placed on a rat bed and inserted in the magnet bore in correspondence with the RF coil center. The mice were anesthetized with a mixture of tiletamine/zolazepam (Zoletil 100; Vibac, Milan, Italy) 20 mg/kg and xylazine (Rompun; Bayer, Milan, Italy) 5 mg/kg. Mice were placed on the surface coil with the tumor area in the middle of the surface coil probe.

Proton T_2_-weighted images were collected using a Turbo RARE pulse sequence (FOV 30 × 30, MTX 128 × 128, slice thickness 3 mm, spatial resolution 0.23 × 0.23 mm^2^, TE 55 ms, TR 2500 ms, RF 12, average 1).

The Na^+^ gradient-echo FLASH sequence was set with the following parameters: FOV 30 × 30, MTX 32 × 32, slice thickness 3 mm, spatial resolution 0.94 × 0.94 mm^2^, TE 2.05 ms, TR 50 ms, average 548.

On the first day experiment, the ^1^H and ^23^Na images were acquired prior to and 2 h after the treatment, which consisted of a Monensin dose of 16 mg/Kg in PBS (freshly prepared from the 22 mM stock solution in DMSO), or of the equivalent vehicle solution for the treated and control groups, respectively. In the following days, the images were acquired 2 h after treatment. At the end of the experiment, the mice were sacrificed (without perfusion) and the Na^+^ content of recovered tumors was quantified, as previously described.

The analysis of the images was performed by using the Paravision 360 preclinical imaging software (Bruker). For each day of the experiment, the mean signal intensity (SI) values were calculated on a region of interest (ROI) manually drawn on the whole tumor and were normalized using the reference sample. The SI change (percentage change) was calculated according to the following equation: percentage change = [(SI_post_ – SI_pre_)]/SI_pre _× 100 where pre refers to the image acquired before the begging of the treatment and post to the image acquired after 2 h from the Monensin treatment. The percentage change, was calculated by averaging the percentage change obtained for mice in the control or treated group, respectively. The millimolar Na^+^ tissue content was expressed as mmol per Kg of tissue, assuming a tissue density to be equal to 1 g/ml.

#### Statistics and reproducibility

Each experiment was repeated independently at least three times. Results are presented as the mean of three-eight independent experiments ±standard deviation. Statistical significance between two groups was determined by unpaired or paired two-tailed Student’s *t* test or Mann–Whitney *U* test. For multiple-group and time-course comparison, one-way ANOVA or two-way ANOVA were used. The distribution normality of all groups was preliminarily verified with the Kolmogorov and Smirnov test. Significance was established at the 5% level. *P* < 0.05 was considered statistically significant (*P < 0.05, **P < 0.01, ***P < 0.001). GraphPad Prism v.8.4.3 (GraphPad Software, San Diego, CA, USA) and OriginPro (Version 2023. OriginLab Corporation, Northampton, MA, USA) were used for statistical analyses.

### Reporting summary

Further information on research design is available in the Nature Portfolio Reporting Summary linked to this article.

## Supplementary information

Supplementary Material

Description of Additional Supplementary Files

Supplementary Data

Supplementary Video 1

Supplementary Video 2

Supplementary Video 3

Supplementary Video 4

Supplementary Video 5

Supplementary Video 6

Supplementary Video 7

Supplementary Video 8

Supplementary Video 9

Reporting Summary

## Data Availability

The numerical source of all data showed in the graphs are included in the Supplementary Data file.
