# Supplementary material for: Boosting intracellular sodium selectively kills hepatocarcinoma cells and induces hepatocellular carcinoma tumor shrinkage in mice
Source: Commun Biol. 2023 May 29;6:574. doi: 10.1038/s42003-023-04946-4 (PMC10227045; doi:10.1038/s42003-023-04946-4)
Supplement: Supplementary file 1 — Supplementary Material [file 42003_2023_4946_MOESM1_ESM.pdf]

Supplementary information

Boosting intracellular sodium selectively kills hepatocarcinoma cells and induces hepatocellular carcinoma tumor shrinkage in mice.

Nausicaa Clemente[1‡], Simona Baroni[2‡], Simone Fiorilla[1], Francesco Tasso[1], Simone Reano[3], Chiara Borsotti[1], Maria Rosaria Ruggiero[2], Elisa Alchera[4], Marco Corrazzari[5], Gillian Walker[1], Antonia Follenzi[1], Simonetta Geninatti Crich[2*] and Rita Carini[1*]

[1]*Department of Health Science Università del Piemonte Orientale, Via Solaroli, 17, 28100 Novara, Italy.*

[2]*Department of Molecular Biotechnology and Health Sciences, University of Torino, Via Nizza, 52, 10126, Torino, Italy*

[3] *Department of Department of Translational Medicine, Unit of Muscle Biology, Università del Piemonte Orientale, Via Solaroli, 17, 28100 Novara, Italy.*

[4] *Division of Experimental Oncology/Unit of Urology, URI, IRCCS, Ospedale San Raffaele, Milan, Italy*

[5]*Department of Health Science and Interdisciplinary Research Center of Autoimmune Disease (IRCAD), Università del Piemonte Orientale, Via Solaroli, 17, 28100 Novara, Italy.*

Correspondence to : rita.carini@med.uniupo.it, simonetta.geninatti@unito.it

REPRESENTATIVE IMAGES from “in vivo” cell living videos of INTRACELLULAR SODIUM and CELL DEATH of C1C7 CELLS EXPOSED OR NOT TO MONENSIN in presence or absence of EXTRACELLULAR Na^+

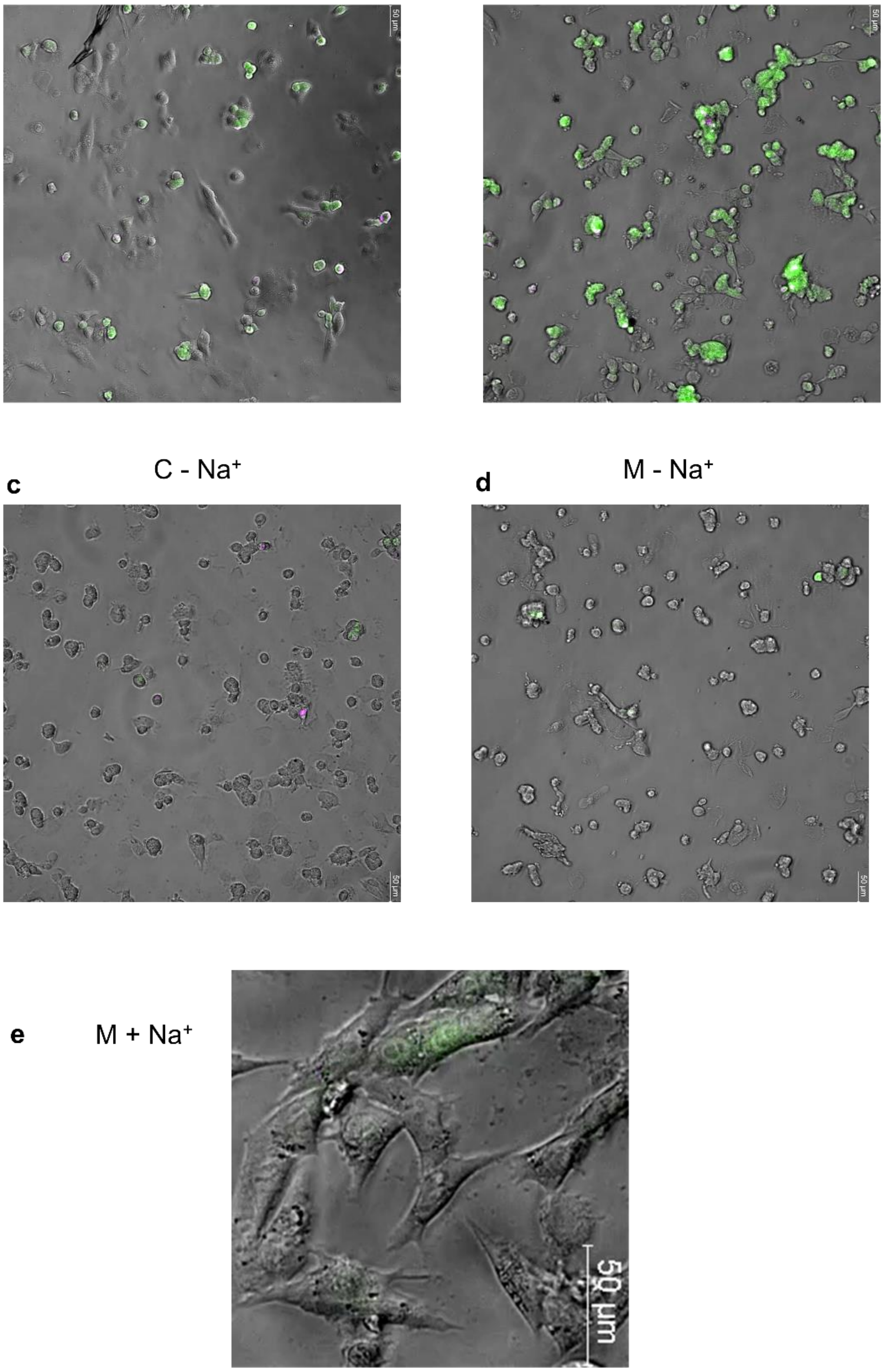

Supplementary Figure 1

Representative *in vivo* cell living images of intracellular Na+ (Green: ION NaTRIUM Green-AM staining) and cell death (Violet, TO-PRO™-3 Iodide staining.

a control C1C7 cell in DMEM medium with Na^+ (image obtained from Supplementary Video 1)

b control C1C7 cell in DMEM medium without Na^+ (image obtained from Supplementary Video 2)

c & e C1C7 cells exposed to Monensin 10 µM in DMEM medium with Na^+ (**c**: image obtained from Supplementary Video 2; **e** shot obtained from Supplementary Video 5)

d C1C7 cells exposed to Monensin 10 µM in DMEM medium without Na^+ (image obtained from Supplementary Video 4)

Magnification: scale bare I=50 µm.

Effects of MONENSIN on FUNCTIONAL Na^+/K^+ ATPase ACTIVITY OF C1C7 cells INCUBATED IN ABSENCE OF EXTRA-CELLULAR Na^+

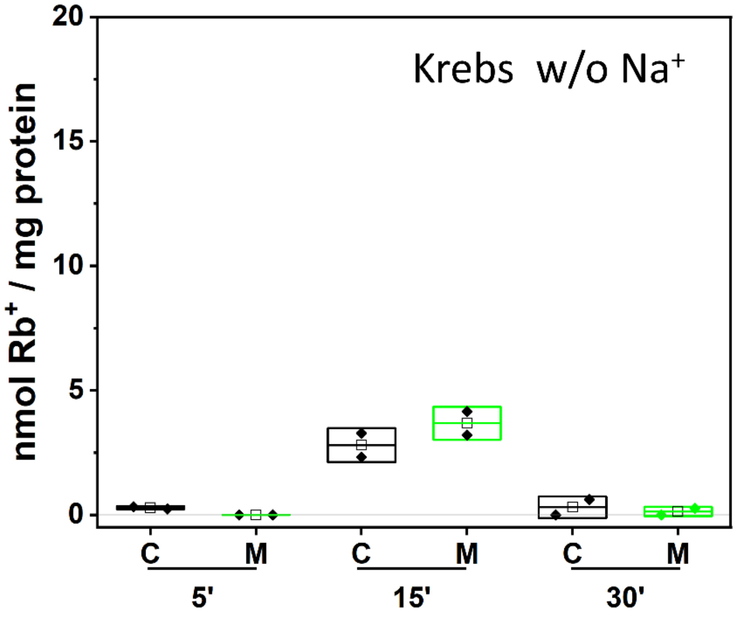

Supplementary Figure 2
Rubidium uptake, on respect to the control (C), for C1C7 cells incubated in Krebs without Na^+ with 10 µM Monensin (M) for 5, 15 or 30 minutes (box: □ mean±standard deviation) ((n=2 independent experiments)

MILLIMOLAR CONCENTRATION of INTRACELLULAR Na^+ OF HEPATOCYTES and HCC CELLS INCUBATED IN PRESENCE OR IN ABSENCE OF EXTRACELLULAR SODIUM WITH OR WITHOUT MONENSIN

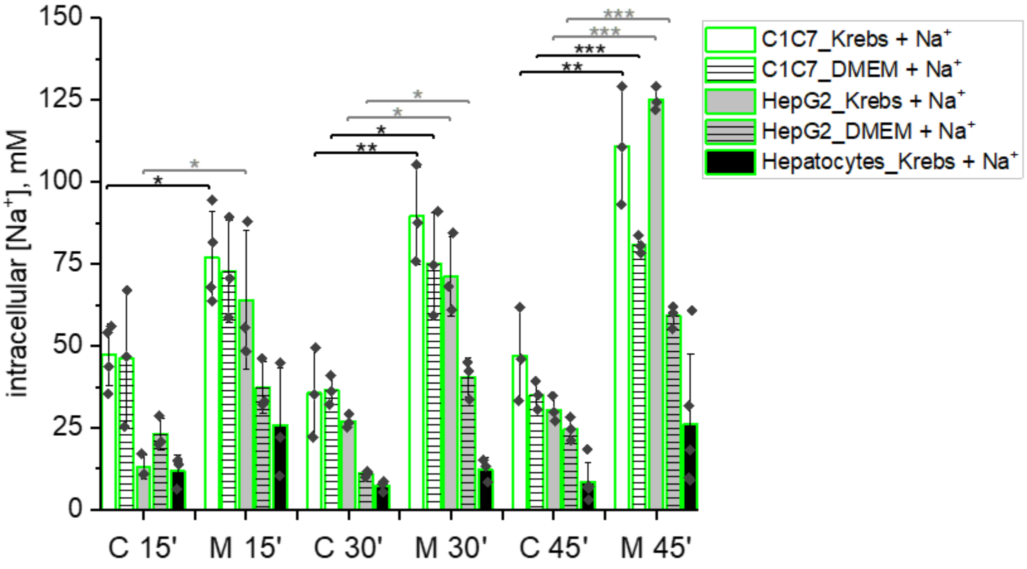

Supplementary Figure 3

Na^+ uptake of C1C7 and HepG2 cells and of hepatocytes (HPs) incubated with (M) or without (C) 10 μM Monensin in Krebs + /- Na^+ or DMEM (culture medium) + Na^+. Bars represent the average and error bar the standard deviation ((n ≥ 3 independent experiments) . *P < 0.05, **P < 0.01, ***P < 0.001 with the Student's t-test.

The nmol of Na^+ per mg of proteins (data in Figure 4A) was converted to a millimolar concentration. Firstly, the data was converted in nmol/cell. The cell number was obtained from the mg of cellular proteins measured by Bradford assay through the calibration curve: [(mg protein)/(number of cells)] obtained by counting the cells before the absorbance measurements. From this calibration, 1 mg of proteins of C1C7, HepG2 and HPs correspond to 3.9, 3 and 0.75 million of cells, respectively. Then, using the cell volume of $5.1x10^{-12}$ L, $3.4x10^{-12}$ L and $2.2x10^{-12}$ L for HP [Junatas KL, Tonar Z, Kubíková T, Liška V, Pálek R, Mik P, Králíčková M, Witter K. Stereological analysis of size and density of hepatocytes in the porcine liver. J Anat. 2017, 230:575-588], HepG2 [Arzumanian VA, Kiseleva OI, Poverennaya EV. The Curious Case of the HepG2 Cell Line: 40 Years of Expertise. Int J Mol Sci. 2021, 22:13135], and C1C7 (calculated using a cell diameter of 13 m), respectively, it was possible to estimate the intracellular Na^+ mM concentration.

REPRESENTATIVE CONFOCAL IMAGES of INTRACELLULAR SODIUM and MITHOCONDRIA of C1C7 cells exposed or not to MONENSIN in presence or absence of EXTRACELLULAR Na$^+$

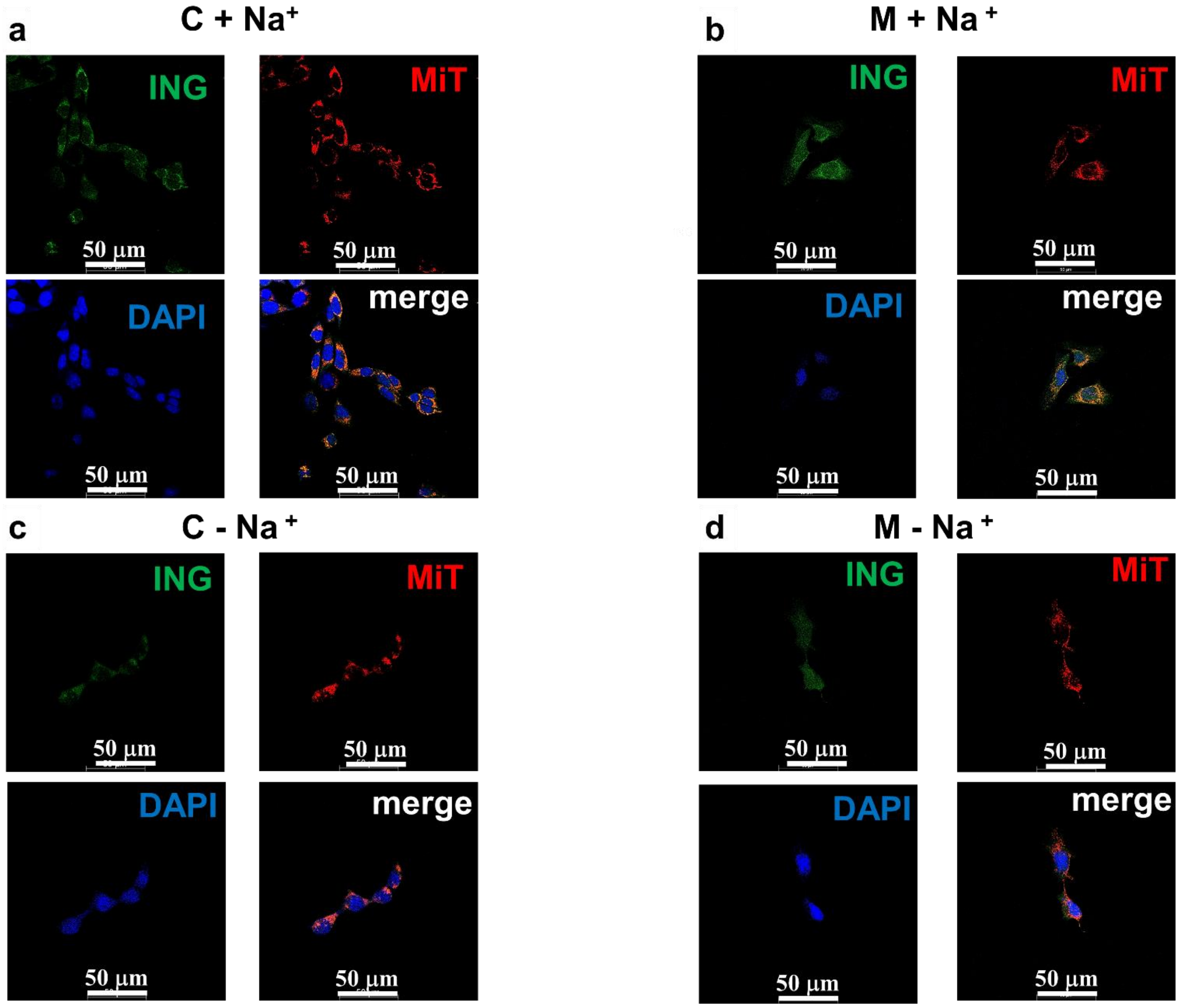

Supplementary Figure 4
Representative confocal images of intracellular Na$^+$ (Green: dye indicator ION NaTRIUM Green-AM, ING) and viable mitochondria (Red: dye indicator MitoTracker Red, MiT) of C1C7 cell exposed (Mon **b**) or not (CTR **a**) 4 h to Monensin 10 μM in DMEM with Na$^+$, or exposed (Mon **d**) or not (CTR **c**) 4 h to Monensin 10 μM in DMEM without Na$^+$ .DAPI (BLU) was to highlight cell nucleus. Single staining and merge are shown. Magnification: scale bar = 50 µm.

TUMOR GROWTH in mice treated or not with 4, 8 or 16 mg/kg of Monensin and ORGAN and MICE WEIGHT of mice treated or not with 8 mg/kg of Monensin

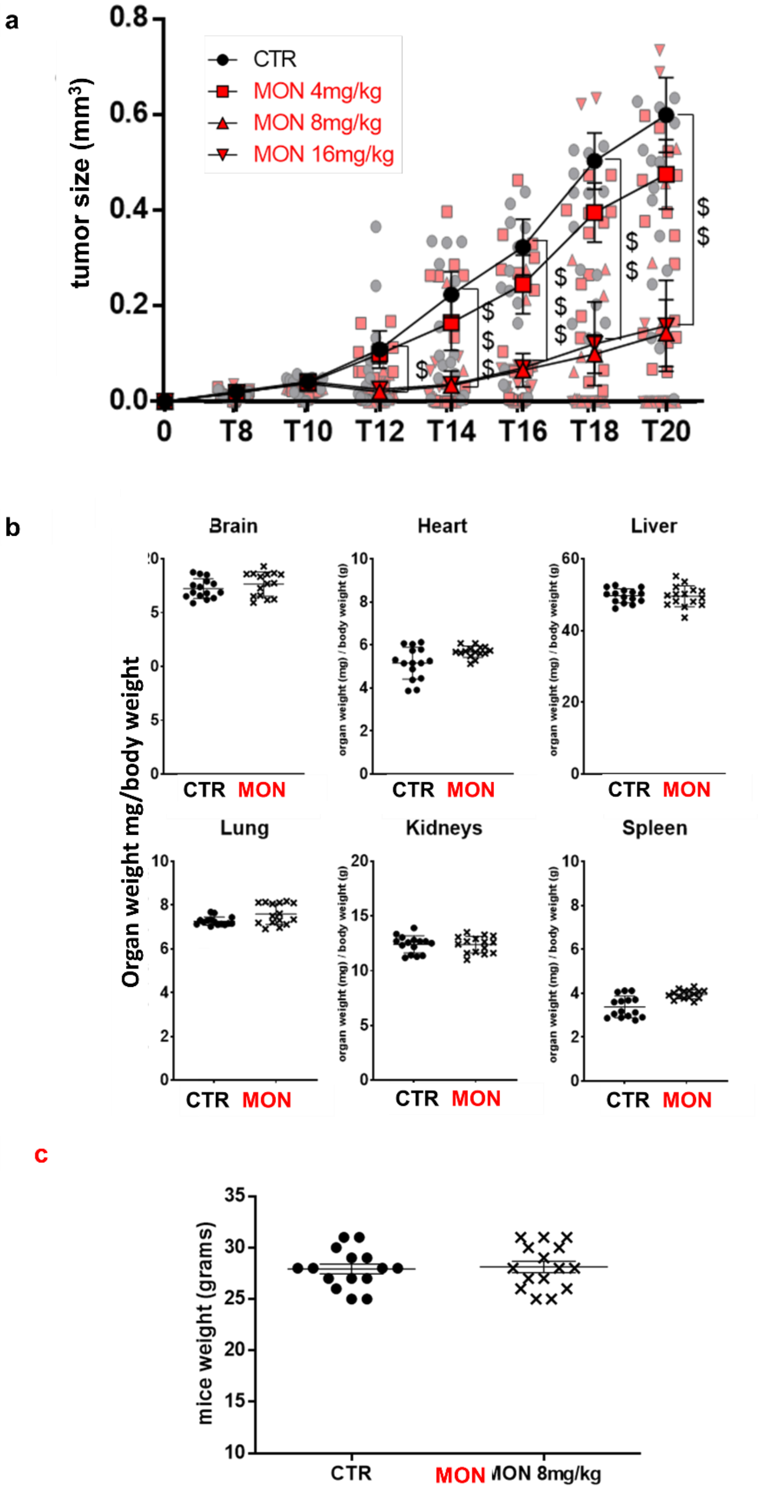

Supplementary Figure 5

a Allograft growth of C1C7 cells in NSG mice i.p. treated with Monensin (4,8 and 16 mg/kg) (MON) or with vehicle (CTR). Symbols represent the average and error bars represent the standard deviation (n=15 CTR and n= 15 M treated mice). P: $< 0.05, $$< 0.01; $$$P < 0.001 by Unpaired T test

b and **c** Organs (b) and mice (c) weight at termination after treatment with Monensin (8 mg/kg) (M) or with vehicle (C). Symbols represent single mice (n=15 CTR and n= 15 M treated mice). Not significant differences between CTR and MON by Unpaired T test

MONENSIN does not affect VITAL ORGANS HYSTOLOGY and PROLIFERATIVE ACTIVITY of INTESTINE and BONE MARROW

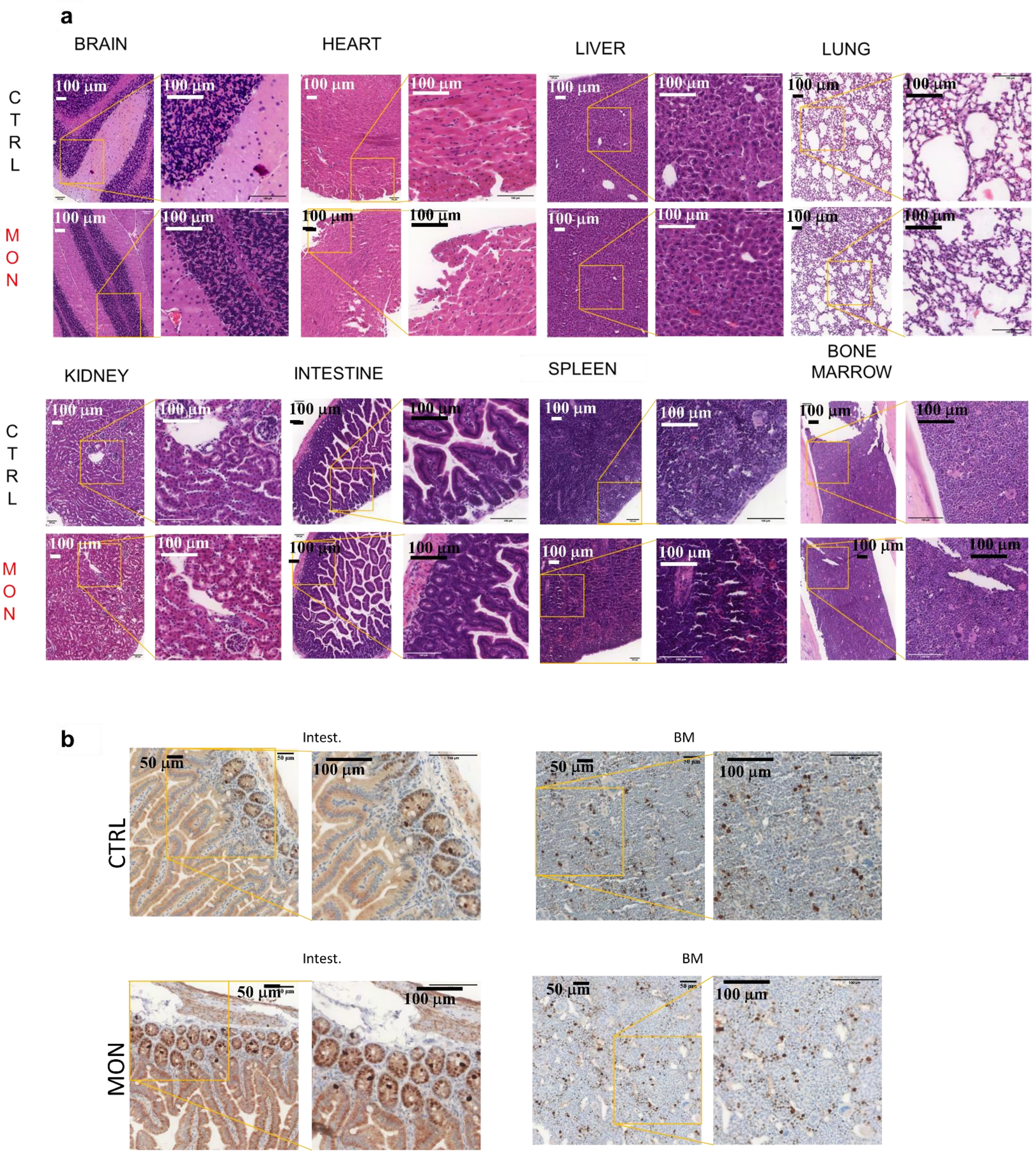

Supplementary Figure 6

a Representative H&E images of healthy tissues of mice i.p. treated with Monensin (8 mg/kg) or vehicle. Magnification: scale bar = 100 µm.

b Representative immunohistochemical staining for ki67 of intestine and bone marrow of mice treated with Monensin or vehicle. Magnification: scale bar = 50 and 100 µm.

.

MONENSIN does NOT AFFECT HEMOPOIESIS in CONTROL MICE

no tumor injection

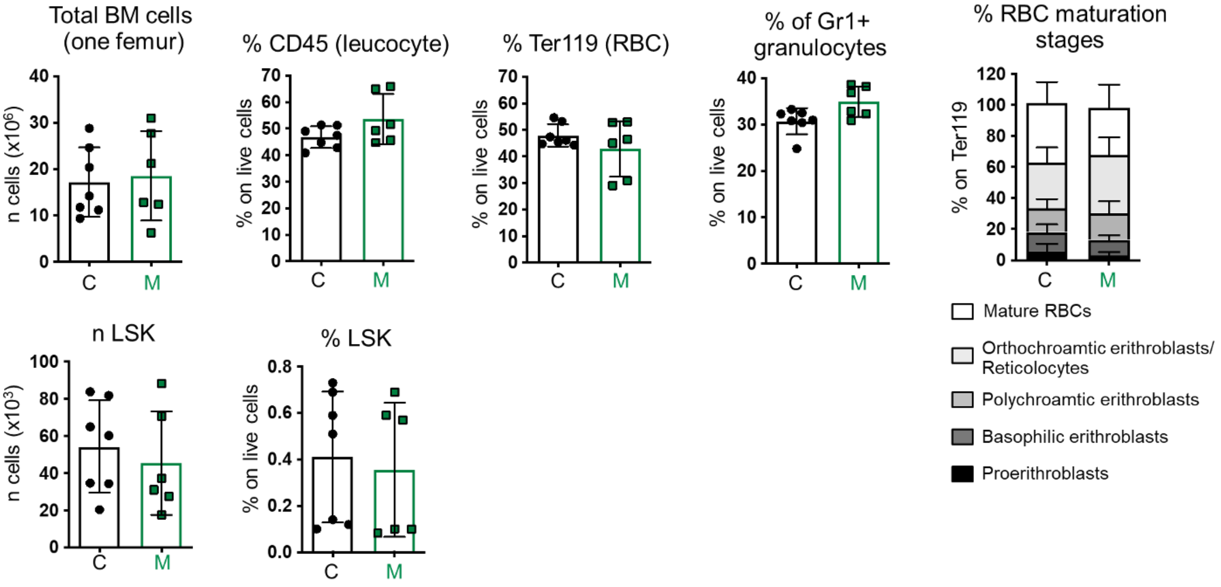

Supplementary Figure 7

Bars plots showing the total number of cells, the percentage of $CD45^+$ leucocytes, of total and relative RBC $Ter119^+$ progenitors, of $Gr1^+$ granulocytes; of number and percentage of LSK in BM of NSG mice without tumor; columns represent the average while bars represent the standard deviation; dots represent single mice, C=control and M=Monensin (n=7 C and n= 6 M treated mice). Difference between C and M groups not significant with unpaired nonparametric Mann Whitney U test; graph with % of red blood cell maturation stage, not significant with two-way ANOVA with a Sidak's multiple comparison test.

GATING STRATEGY OF FIGURE 7g and of Supplementary Figure 7

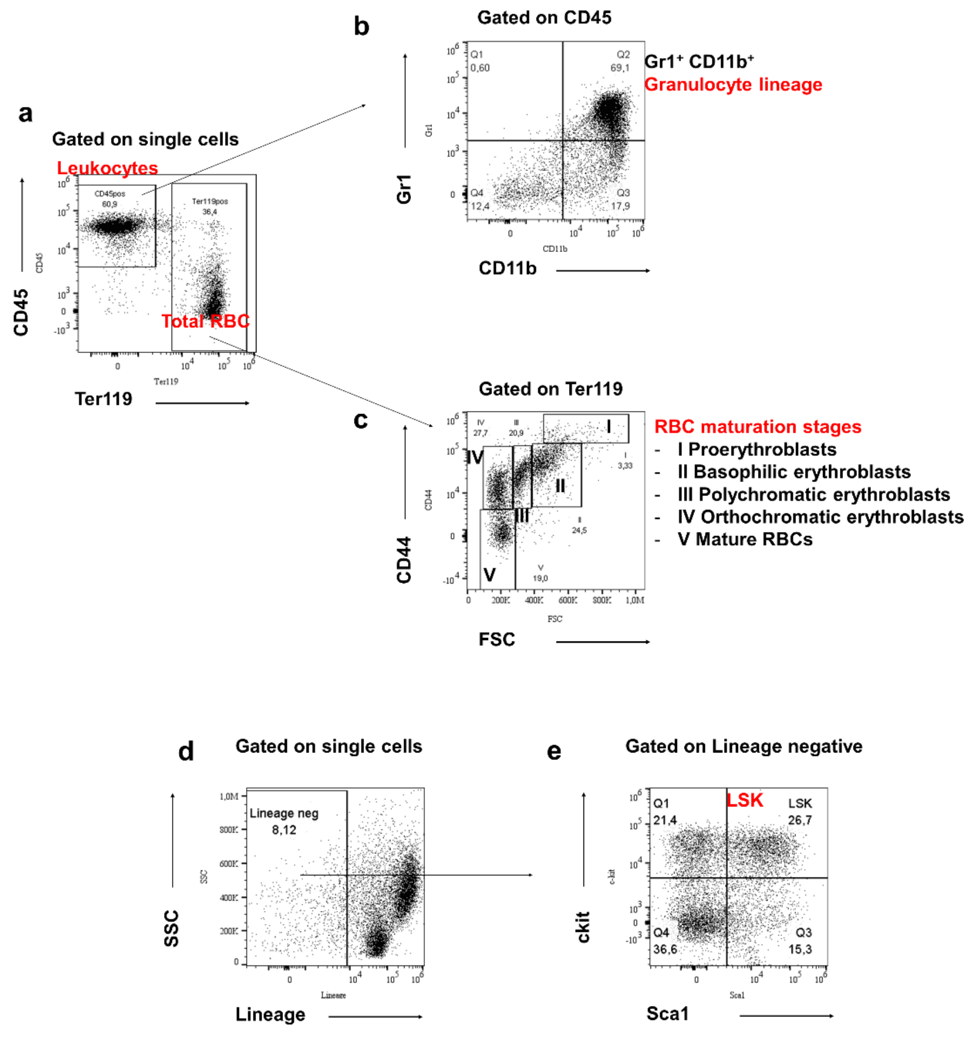

Lineage = Gr1, CD11b, B220, CD3, Ter119

Supplementary Figure 8

Representative dot plots showing the gating strategy for identifying the listed bone marrow (BM) populations (**a, b, c**) and for identifying the mouse hematopoietic stem (LSK) cells in BM (**d, e**).

a Identification of $CD45^+$ leukocytes and total $Ter119^+$ cells.
b Identification of $Gr1^+CD11b^+$ granulocytes.
c Identification of the 5 red blood cells (RBC) maturation stages.
d Identification of the lineage (Gr1, CD11b, B220, CD3, Ter119) negative cell population.
e Identification of $ckit^+$ $Sca1^+$ cells.

INTRATUMOUR Na^+ CONCENTRATION EVALUATED BY ^{23}Na-MRI

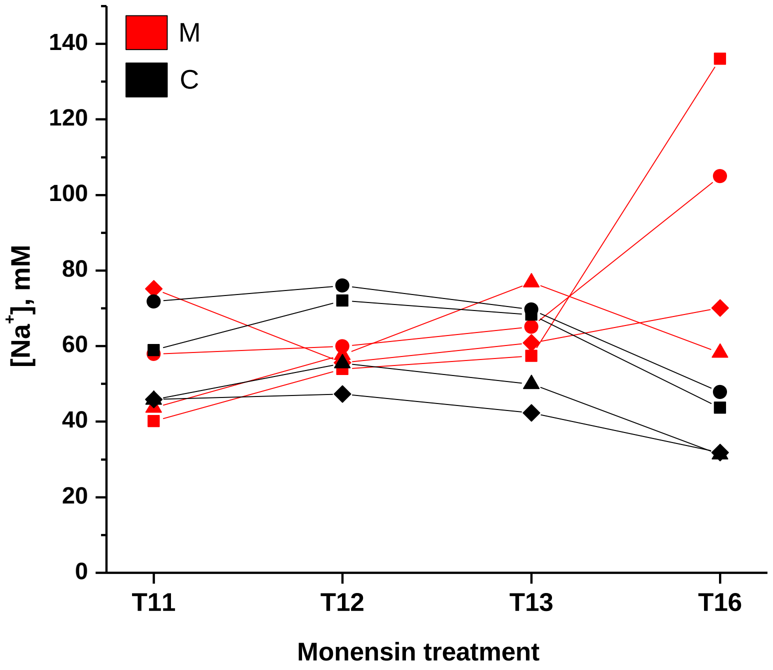

Supplementary Figure 9

Intra-tumour Na^+ tissue concentration, extrapolated from the ^{23}Na-MRI images (using the 75 mM Na^+ reference) for the Monensin treated (M) and control (C) mice. Symbols represent different mice (n ≥ 3 C, n≥ 3 M treated mice). The time (days) after the inoculation of C1C7 cells is indicated on the x axes.

HYPERINTENSE TUMOUR AREAS EVALUATED BY ^{23}Na-MRI

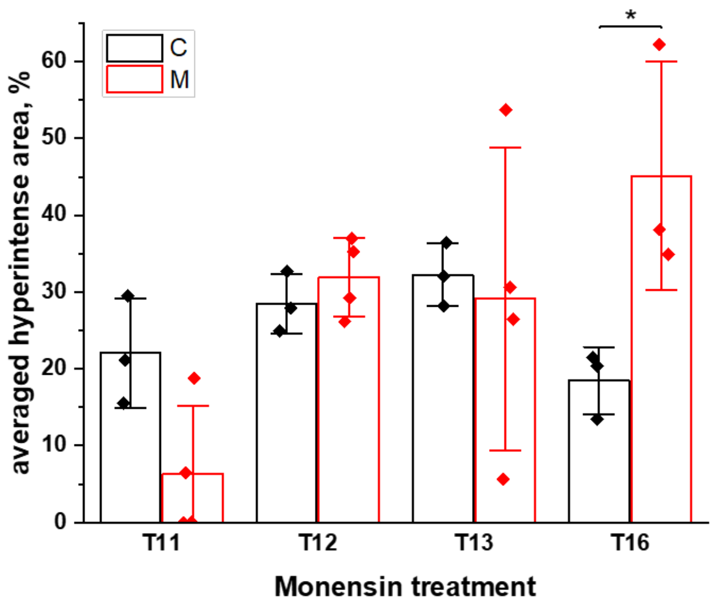

Supplementary Figure 10: Averaged percentage of the hyperintense area of the tumor of Monensin treated (M) and control (C) mice with respect to the whole tumour area, calculated from the ^{23}Na MRI images. Bars represent the average and error bars the standard deviation (n ≥ 3 C, n≥ 3 M treated mice).The time (days) after the inoculation of C1C7 cells is indicated on the x axes. *P < 0.05 with Student ‘t test.

Supplementary Table 1

Correlation between the Na^+ tissue content determined by MRI and ICP measurements.

Mouse group	[Na^+], mmol / Kg tissue*	
	From ^{23}Na MRI data	From ICP-MS data
Monensin treated	70.1	79.3
	136.1	129.4
	58.3	60.3
Control	43.7	57.9
	47.8	56.5
