# Supplementary material for: Boosting intracellular sodium selectively kills hepatocarcinoma cells and induces hepatocellular carcinoma tumor shrinkage in mice
Source: Commun Biol. 2023 May 29;6:574. doi: 10.1038/s42003-023-04946-4 (PMC10227045; doi:10.1038/s42003-023-04946-4)
Supplement: Supplementary file 2 — Description of Additional Supplementary Files [file 42003_2023_4946_MOESM2_ESM.pdf]

Description of Additional Supplementary Files

File name: Supplementary Data
Description: All source data underlying the graphs in the main figures and in the supplementary figures.

File name: Supplementary Video 1
Description: Representative *in vivo* cell living video of intracellular Na+ (Green: ION NaTRIUM Green-AM staining) and cell death (Violet, TO-PRO™-3 Iodide staining) of control C1C7 cell in DMEM medium with Na^+

File name: Supplementary Video 2
Description: Representative *in vivo* cell living video of intracellular Na+ (Green: ION NaTRIUM Green-AM staining) and cell death (Violet, TO-PRO™-3 Iodide staining) of C1C7 cell exposed to Monensin 10 µM in DMEM medium with Na^+

File name: Supplementary Video 3

Description: Representative *in vivo* cell living video of intracellular Na+ (Green: ION NaTRIUM Green-AM staining) and cell death (Violet, TO-PRO™-3 Iodide staining) of control C1C7 cell in DMEM medium without Na^+

File name: Supplementary Video 4
Description: Representative *in vivo* cell living video of intracellular Na+ (Green: ION NaTRIUM Green-AM staining) and cell death (Violet, TO-PRO™-3 Iodide staining) of C1C7 cell exposed to Monensin 10 µM in DMEM medium without Na^+

File name: Supplementary Video 5
Description: Representative *in vivo* cell living Video of intracellular Na^+ (Green: ION NaTRIUM Green-AM staining) and cell death (Violet, TO-PRO™-3 Iodide staining, TO-PRO3) of C1C7 cell exposed to Monensin 10 µM in DMEM medium with Na^+ (from this video were obtained the image of Figure 1c)

File name: Supplementary Video 6
Description: Z-stack video of C1C7 cell (stained with ION NaTRIUM Green-AM, ING, MiTo Tracker, MiT and DAPI) exposed 4 h to 10 µM Monensin (M) in DMEM + Na^+. Z start: 4681,0974 µm, Z end: 4687,7356 µm. 24 step, 1 step = 0,2886 µm. DAPI staining

File name: Supplementary Video 7
Description: Z-stack video of C1C7 cell (stained with ION NaTRIUM Green-AM, ING, MiTo Tracker, MiT and DAPI) exposed 4 h to 10 µM Monensin (M) in DMEM + Na^+. Z start: 4681,0974 µm, Z end: 4687,7356 µm. 24 step, 1 step = 0,2886 µm. ING staining.

File name: Supplementary Video 8

Description: Z-stack video of C1C7 cell (stained with ION NaTRIUM Green-AM, ING, MiTo Tracker, MiT and DAPI) exposed 4 h to 10 µM Monensin (M) in DMEM + Na^{+}. Z start: 4681,0974 µm, Z end: 4687,7356 µm. 24 step, 1 step = 0,2886 µm. MiT staining.

File name: Supplementary Video 9

Description: Z-stack video of C1C7 cell (stained with ION NaTRIUM Green-AM, ING, MiTo Tracker, MiT and DAPI) exposed 4 h to 10 µM Monensin (M) in DMEM + Na^{+}. Z start: 4681,0974 µm, Z end: 4687,7356 µm. 24 step, 1 step = 0,2886 µm. Merge DAPI, ING and MiT.
