# Supplementary material for: Boosting intracellular sodium selectively kills hepatocarcinoma cells and induces hepatocellular carcinoma tumor shrinkage in mice
Source: Commun Biol. 2023 May 29;6:574. doi: 10.1038/s42003-023-04946-4 (PMC10227045; doi:10.1038/s42003-023-04946-4)
Supplement: Supplementary file 13 — Reporting Summary [file 42003_2023_4946_MOESM13_ESM.pdf]

nature portfolio

Corresponding author(s): Rita Carini
Simonetta Geninatti Crich

Last updated by author(s): May 12, 2023

Reporting Summary

Nature Portfolio wishes to improve the reproducibility of the work that we publish. This form provides structure for consistency and transparency in reporting. For further information on Nature Portfolio policies, see our Editorial Policies and the Editorial Policy Checklist.

Statistics

For all statistical analyses, confirm that the following items are present in the figure legend, table legend, main text, or Methods section.

n/a	Confirmed	
☐	☒	The exact sample size (*n*) for each experimental group/condition, given as a discrete number and unit of measurement
☒	☐	A statement on whether measurements were taken from distinct samples or whether the same sample was measured repeatedly
☐	☒	The statistical test(s) used AND whether they are one- or two-sided<br>*Only common tests should be described solely by name; describe more complex techniques in the Methods section.*
☒	☐	A description of all covariates tested
☒	☐	A description of any assumptions or corrections, such as tests of normality and adjustment for multiple comparisons
☐	☒	A full description of the statistical parameters including central tendency (e.g. means) or other basic estimates (e.g. regression coefficient) AND variation (e.g. standard deviation) or associated estimates of uncertainty (e.g. confidence intervals)
☐	☒	For null hypothesis testing, the test statistic (e.g. *F*, *t*, *r*) with confidence intervals, effect sizes, degrees of freedom and *P* value noted<br>*Give P values as exact values whenever suitable.*
☒	☐	For Bayesian analysis, information on the choice of priors and Markov chain Monte Carlo settings
☒	☐	For hierarchical and complex designs, identification of the appropriate level for tests and full reporting of outcomes
☒	☐	Estimates of effect sizes (e.g. Cohen's *d*, Pearson's *r*), indicating how they were calculated

Our web collection on statistics for biologists contains articles on many of the points above.

Software and code

Policy information about availability of computer code

Data collection	Histological and immunohystochemical analysis: cell-detection conducted using QuPath's built-in “Positive cell detection” [Bankhead P, Loughrey MB, Fernández JA, Dombrowski Y, McArt DG, Dunne PD et al QuPath: Open source software for digital pathology image analysis. Sci Rep. 2017;7:16878]]
Data analysis	analysis of samples acquired with Attune NxT Acoustic Focusing Cytometer (Thermo Fisher Scientific): FlowJo v10 software (BD Biosciences). Histological and immunohystochemical analysis: cell-detection conducted using QuPath's built-in “Positive cell detection” Software version: QuPath-0.3.2

For manuscripts utilizing custom algorithms or software that are central to the research but not yet described in published literature, software must be made available to editors and reviewers. We strongly encourage code deposition in a community repository (e.g. GitHub). See the Nature Portfolio guidelines for submitting code & software for further information.

Data

Policy information about availability of data

All manuscripts must include a data availability statement. This statement should provide the following information, where applicable:

- Accession codes, unique identifiers, or web links for publicly available datasets
- A description of any restrictions on data availability
- For clinical datasets or third party data, please ensure that the statement adheres to our policy

All data generated or analysed during this study are included in this published article (and its supplementary information files)

Human research participants

Policy information about studies involving human research participants and Sex and Gender in Research.

Reporting on sex and gender	*Use the terms sex (biological attribute) and gender (shaped by social and cultural circumstances) carefully in order to avoid confusing both terms. Indicate if findings apply to only one sex or gender; describe whether sex and gender were considered in study design whether sex and/or gender was determined based on self-reporting or assigned and methods used. Provide in the source data disaggregated sex and gender data where this information has been collected, and consent has been obtained for sharing of individual-level data; provide overall numbers in this Reporting Summary. Please state if this information has not been collected. Report sex- and gender-based analyses where performed, justify reasons for lack of sex- and gender-based analysis.*
Population characteristics	*Describe the covariate-relevant population characteristics of the human research participants (e.g. age, genotypic information, past and current diagnosis and treatment categories). If you filled out the behavioural & social sciences study design questions and have nothing to add here, write "See above."*
Recruitment	*Describe how participants were recruited. Outline any potential self-selection bias or other biases that may be present and how these are likely to impact results.*
Ethics oversight	*Identify the organization(s) that approved the study protocol.*

Note that full information on the approval of the study protocol must also be provided in the manuscript.

Field-specific reporting

Please select the one below that is the best fit for your research. If you are not sure, read the appropriate sections before making your selection.

- [x] Life sciences
- [ ] Behavioural & social sciences
- [ ] Ecological, evolutionary & environmental sciences

For a reference copy of the document with all sections, see nature.com/documents/nr-reporting-summary-flat.pdf

Life sciences study design

All studies must disclose on these points even when the disclosure is negative.

Sample size	The sample sizes employed were the same reported in already estabilished methods reported in previous published (and cited) articles.
Data exclusions	Data were excluded from analysis only in the occasions of experiments clearly aborted from the beginnings (i.e. viability of control cells very low from the start of the treatment)
Replication	The experiments were replicated from 3 to 5 times. The experiments that failed to reproduce the data were that described in the previous point.
Randomization	Randomization was applied to the "in vivo" studies: i.e. among the group of tumor bearing mice, the sub-group of mice that were treated with the drug were chosen randomly in the tumor bearing group, proving that the size of the tumors were almost the same in the control and treated group before the beginning of the treatment
Blinding	Blinding was applied to the "in vivo" manual determination of tumor sizes: in this case the investigator measuring the size of the tumor did not know from which group (control or treated) belonged the mouse

Reporting for specific materials, systems and methods

We require information from authors about some types of materials, experimental systems and methods used in many studies. Here, indicate whether each material, system or method listed is relevant to your study. If you are not sure if a list item applies to your research, read the appropriate section before selecting a response.

Materials & experimental systems

n/a	Involved in the study
[ ]	[x] Antibodies
[x]	[ ] Eukaryotic cell lines
[x]	[ ] Palaeontology and archaeology
[ ]	[x] Animals and other organisms
[x]	[ ] Clinical data
[x]	[ ] Dual use research of concern

Methods

n/a	Involved in the study
[x]	[ ] ChIP-seq
[ ]	[x] Flow cytometry
[x]	[ ] MRI-based neuroimaging

Antibodies

Antibodies used

CD11b mouse PE M1/70 ThermoFisher
CD3 mouse PE 145-2C11 ThermoFisher
Ter119 mouse PE TER-119 ThermoFisher
Gr1
(Ly-6G/Ly-6C) mouse PE-Cy5.5 RB6-8C5 ThermoFisher
Gr1
(Ly-6G/Ly-6C) mouse PE RB6-8C5 ThermoFisher
Sca1 mouse PE-Cy7 D7 ThermoFisher
CD44 mouse APC IM7 ThermoFisher
CD117 (c-kit) mouse APC 2B8 ThermoFisher
B220 mouse PE RA3-6B2 ThermoFisher
CD45 mouse APC-eFluor780 30-F11 ThermoFisher

Validation

data provided in the manuscript

Animals and other research organisms

Policy information about studies involving animals; ARRIVE guidelines recommended for reporting animal research, and Sex and Gender in Research

Laboratory animals

NOD.Cg-PrkdcscidIl2rgtm1Wjl/SzJ (Jackson stock No 005557) (NSG) mice

Wild animals

Provide details on animals observed in or captured in the field; report species and age where possible. Describe how animals were caught and transported and what happened to captive animals after the study (if killed, explain why and describe method; if released, say where and when) OR state that the study did not involve wild animals.

Reporting on sex

Six-to-eight-week-old adult male NSG mice.

Field-collected samples

Mice were mantained under under pathogen-free conditions in the animal facility of Università del Piemonte Orientale (UPO), Department of Health Sciences, with free access to water and food, controlled temperature and standardized photoperiod.

Ethics oversight

treated in accordance with the University of Piemonte Orientale (UPO), Novara, Italy Ethical Committee and European guidelines (Experimental protocol authorization n. 851/2020-PR, released in 19/08/2020 from Italian Ministry of Health for protocol n. DB064.60)

Note that full information on the approval of the study protocol must also be provided in the manuscript.

Flow Cytometry

Plots

Confirm that:

- [x] The axis labels state the marker and fluorochrome used (e.g. CD4-FITC).
- [x] The axis scales are clearly visible. Include numbers along axes only for bottom left plot of group (a 'group' is an analysis of identical markers).
- [x] All plots are contour plots with outliers or pseudocolor plots.
- [x] A numerical value for number of cells or percentage (with statistics) is provided.

Methodology

Sample preparation	One femur per mouse was collected and maintained in cold RPMI 1640 medium supplemented of 5% FBS. BM cells were harvested by flushing the bones with a 26G needle and passed through 40 micronm cell filter to obtain single cells suspension.
Instrument	Attune NxT Acoustic Focusing Cytometer (Thermo Fisher Scientific)
Software	FlowJo v10 software (BD Biosciences).
Cell population abundance	Samples were stained with fluorochrome-labeled monoclonal antibodies (reported in the previous section and in Supplementary materials) against mouse markers. Master mix of antibodies was made for each staining in FACS buffer, cells were washed and resuspended in master mix and incubated for 15 min at 4°C. Samples were then acquired on the Attune NxT Acoustic Focusing Cytometer
Gating strategy	Gating strategy based on following paper:<br>Ke Chen et al. PNAS 2009;106:41:17413-17418. Dot plots are not included in the Supplementary Informations at this stage but will be addedd if requested.

☒ Tick this box to confirm that a figure exemplifying the gating strategy is provided in the Supplementary Information.
